# QLSA-MOEAD integration for precision task scheduling in heterogeneous computing environments

**DOI:** 10.1038/s41598-026-36916-1

**Published:** 2026-02-17

**Authors:** Abla Saad, Osama Abd el-Raouf, Mohiy Hadhoud, Ahmed Kafafy

**Affiliations:** 1 Faculty of AI, Machine Intelligence Dept, Minufiya University, Shebeen El-Kom, Egypt; 2 Faculty of Computers and Information, Information Technology Dept, Minufiya University, Shebeen El-Kom, Egypt; 3 Faculty of AI, Data Science Dept, Minufiya University, Shebeen El-Kom, Egypt

**Keywords:** Heterogeneous Computing Environment, DAG (Directed, MOEA/D, Q-Learning, Simulated Annealing, Engineering, Mathematics and computing

## Abstract

Heterogeneous computing infrastructures integrating CPUs, GPUs, and FPGAs present critical challenges in efficient task scheduling due to hardware diversity, complex task dependencies, and conflicting optimization objectives. This work formulates workflow scheduling as a multi-objective optimization problem that minimizes makespan and maximizes resource utilization. For synthetic benchmarks (FFT, Molecular), the approach minimizes makespan and maximizes resource utilization. For the CyberShake seismic workflow, energy consumption is added as a third objective. This research proposes QLSA-MOEAD, a hybrid framework combining three complementary mechanisms: Q-learning for intelligent initialization, Simulated Annealing for local refinement, and MOEA/D for multi-objective decomposition. This integration balances exploration and exploitation effectively. Comprehensive evaluations on 20 test cases (structured FFT, unstructured molecular, and real-world CyberShake workflows) show superior performance. QLSA-MOEAD achieves the best solution quality in 14 out of 16 FFT/molecular cases and outperforms all baselines on CyberShake. A large-scale Montage workflow (100 tasks, 179 dependencies) validates scalability under real-time task arrivals. The framework maintains excellent convergence and diversity across different CCR levels. Q-learning achieves fast decision-making with 0.80–1.70 ms response time. Statistical validation (Wilcoxon and Friedman tests), ablation studies, and parameter sensitivity analysis confirm framework robustness. These results establish QLSA-MOEAD as an effective solution for both static and dynamic workflow scheduling in heterogeneous environments.

## introduction

Heterogeneous computing systems combine different processor types-CPUs, GPUs, FPGAs, TPUs, and ASICs-to meet the demands of data-intensive applications. Each processor type serves a specific role: CPUs handle general tasks, GPUs accelerate parallel computations, and FPGAs provide customizable hardware logic. TPUs target deep learning workloads, while ASICs deliver optimized performance for specialized applications like blockchain and real-time analytics^1^. These systems distribute work across specialized units to improve both throughput and energy efficiency^2^.

These systems are commonly deployed on cloud platforms, high-performance clusters, and grid infrastructures. Many applications in scientific computing and industrial processing use Directed Acyclic Graphs (DAGs) to represent workloads, where nodes represent tasks and edges capture dependencies^3–6^. Task scheduling in these environments is NP-hard. It requires balancing. multiple goals: reducing total completion time (makespan), distributing load evenly across processors, and in some cases minimizing energy use. Scheduling methods fall into two categories: Static approaches assign tasks before execution begins, while dynamic methods adjust assignments. at runtime^7,8^. However, many dynamic techniques start from random initial solutions, which slows convergence and can reduce solution quality. The main contributions of this work can be summarized as follows:

- **Enhanced multi-objective scheduling:** This study proposes an advanced scheduling framework for heterogeneous systems to optimize multiple objectives simultaneously. The framework formulates workflow scheduling as a multi-objective optimization problem. For synthetic benchmarks (FFT, Molecular), it minimizes makespan and maximizes resource utilization. For the real-world CyberShake seismic workflow, it extends to three-objective optimization by incorporating energy consumption.
- **Integration of MOEA/D optimization:** The proposed framework builds upon the Multi-Objective Evolutionary Algorithm based on Decomposition (MOEA/D), which is highly effective for multi-objective optimization challenges. Instead of combining objectives into a single scalar function, MOEA/D decomposes the problem into several subproblems using evenly spaced weight vectors. A neighborhood-based information exchange mechanism preserves solution diversity and enhances search performance to yield well-distributed Pareto-optimal solutions.
- **Reinforcement learning-driven scheduling:** Reinforcement learning has proven effective for complex decision-making tasks. This work employs Q-learning as the core mechanism for schedule construction in heterogeneous computing environments. Rather than relying on random task allocation, the proposed algorithm learns optimal task-processor mappings through accumulated experience and dynamically adapts to workflow characteristics for more efficient and intelligent scheduling. Once the knowledge-driven schedule is generated, Simulated Annealing (SA) is applied as a post-optimization stage to escape local optima and further refine performance. This two-stage process forms the basis of the proposed QLSA-MOEA/D framework to accelerate convergence and improve the overall quality of obtained schedules. Beyond its effectiveness in static environments, the framework efficiently adapts to system variations and real-time changes in task execution conditions, making it highly suitable for dynamic workflows.
- **Comprehensive validation:** The framework is validated through extensive experiments on 20 test cases. These include 16 synthetic cases (FFT, Molecular) for two-objective optimization and 4 real-world cases (CyberShake) for three-objective optimization with energy consumption. Wilcoxon signed-rank and Friedman tests confirm statistical significance. Ablation studies isolate the contribution of each component (Q-learning, simulated annealing, and MOEA/D). Parameter sensitivity analysis on Q-learning hyperparameters (learning rate, discount factor, and exploration rate) demonstrates robustness.

The structure of this paper is arranged to ensure clarity and logical flow. Section 2 presents an overview of the related studies. Section 3 explains the system model and the main notations summarized in Table 1. Section 4 describes the proposed methodology in detail. Section 5 outlines the evaluation setup and performance metrics. Section 6 examines the convergence behavior and computational complexity. Section 7 reports the experimental results, followed by a detailed discussion in Section 8. Finally, Section 9 summarizes the main conclusion and highlights possible directions for future research.

**Table 1:** Notation used in the scheduling model.

Notation	Description
$E_d$	Set of directed links (Edges) defining task dependencies.
*T*	Total tasks forming the workflow.
*P*	Number of heterogeneous processors.
$t_i$	Identifier for task *i*.
$P_x$	Identifier for processor *x*.
$T_{\text {entry}}$	Task that initiates the workflow.
$T_{\text {exit}}$	Task that ends the workflow.
$\text {Succ}(t_i)$	Tasks executed after $t_i$.
$\text {Pred}(t_i)$	Tasks executed before $t_i$.
$W(t_i, p_j)$	Computation cost of $t_i$ on $p_j$.
$\overline{W(t_i)}$	Average computation cost of $t_i$ over all processors.
$C(t_i, t_j)$	Communication time between $t_i$ and $t_j$.
$EST(t_i, P_x)$	Earliest start time of $t_i$ on $P_x$.
$EFT(t_i, P_x)$	Earliest completion time of $t_i$ on $P_x$.
$\text {Avail}(P_x)$	Earliest availability of $P_x$.
$AST(t_i, P_x)$	Actual starting time of $t_i$ on $P_x$.
$AFT(t_i, P_x)$	Actual completion time of $t_i$ on $P_x$.
CCR	Communication to computation ratio metric.
*O*	Output solutions from optimization process.
*R*	Reference solution set for evaluation.
*Popsize*	Population size in MOEA/D.
*MaxGen*	Maximum Number of generations in MOEA/D.
*I*	Iterations of Simulated Annealing or Tabu Search
$E_{ql}$	Episodes in Q-learning training.

## Related work

Task scheduling in heterogeneous computing has received significant attention. Researchers focus on improving system throughput, balancing workloads, and reducing energy use. These environments integrate diverse processing units such as CPUs, GPUs, DSPs, ASICs, and FPGAs. Each unit has distinct architectures and instruction sets for cooperative execution of computational workloads ^9,10^. GPU–CPU-based platforms have attracted significant attention in high-performance computing due to their high parallel efficiency and cost-effectiveness. However, designing scalable and efficient scheduling algorithms for such heterogeneous systems remains a challenging and open problem ^11^.

A common representation for scheduling problems in these environments is the Directed Acyclic Graph (DAG). In this model, tasks are represented as nodes, and data or execution dependencies are represented as edges. DAG-based modeling supports optimization across multiple objectives. These include makespan minimization, energy reduction, and improved resource utilization through parallel execution. This approach is widely adopted in scientific workflows, cloud computing, and large-scale analytics^6,12^. Despite its flexibility, DAG-based modeling depends heavily on the efficiency of the underlying scheduling strategy.

Task scheduling in heterogeneous computing environments has been widely investigated due to its crucial role in optimizing system performance and resource utilization. While existing research provides valuable foundations, critical analysis reveals persistent limitations in adaptivity, multi-objective optimization, and scalability. Early algorithms such as Heterogeneous Earliest Finish Time (HEFT) and Critical Path on a Processor (CPOP)^13^ established benchmarks for static scheduling. HEFT prioritizes task execution based on rank values to minimize makespan, while CPOP focuses on the critical path. However, both assume static conditions and lack mechanisms to adapt to dynamic workload or resource changes typical in real heterogeneous systems.

The NP-hard nature of scheduling problems has led to the use of metaheuristics such as GRASP^14,15^, Simulated Annealing^16^, and Tabu Search^17–19^. These algorithms explore large solution spaces but face two major challenges: high computational cost that limits scalability and strong sensitivity to parameter tuning that affects robustness. Although GRASP has shown competitive results in minimizing completion time^20^, most metaheuristics remain focused on single-objective optimization, overlooking essential concerns such as energy efficiency and reliability.

Recent research has increasingly targeted multiple objectives but faces limitations. Shirvani et al.^21^ proposed a bi-objective Simulated Annealing algorithm for makespan and cost minimization but optimized each objective independently. Akbari et al.^22^ combined Cuckoo Optimization with GA but relied on weighted-sum aggregation, which imposes fixed preference weights and prevents full Pareto front discovery. Similarly, hybrid approaches such as the Grey Wolf Optimizer with GA^23^ and NSGA-III implementations^24^ improved convergence yet maintained high computational complexity and lacked adaptability.

Hosseini’s comprehensive survey^25^ provides a valuable taxonomy of scheduling algorithms, summarized in Table 2. However, it focuses primarily on structural classification rather than critical evaluation of adaptivity. The taxonomy shows that while machine learning methods offer dynamic adjustment, they require extensive training data, whereas hybrid methods balance exploration and exploitation but remain complex. Most existing algorithms exhibit limited adaptivity to dynamic changes in real environments.

**Table 2:** Summary of scheduling algorithm categories (adapted and extended from Hosseini^25^).

Aspect	List Scheduling	Heuristic	Metaheuristic	Machine Learning	Multi-Objective	Hybrid
**Main Strategy**	Priority-based ordering	Rule-based allocation or clustering	Global population or neighborhood search	Predictive or RL-based task–resource mapping	Trade-off among multiple goals	Combined global and local search
**Adaptivity**	No	Limited	Partial	Yes	Partial	Yes
**Learning-based**	No	No	No	Yes	No	Optional
**Advantages**	Simple and fast; suitable for static cases	Fast decisions; handles medium-size workflows	Solves NP-hard problems; explores large search spaces	Adapts to dynamic environments; real-time response	Produces Pareto-optimal fronts	Balances exploration and exploitation; improved convergence
**Limitations**	Cannot adapt to dynamic changes; near-optimal only	Approximate; lacks global search	High computational cost; parameter sensitivity	Requires large datasets; complex training	Computational intensive; parameter tuning	Complex design; higher runtime
**Objective Type**	Single	Single	Single/Multi	Single/Multi	Multi	Single/Multi

While Hosseini’s taxonomy offers a comprehensive overview, most listed methods remain static or semi-adaptive and rely on predefined rules instead of intelligent feedback mechanisms. Their performance degrades under dynamic workloads. In contrast, the proposed QLSA-MOEAD framework combines reinforcement learning with metaheuristic optimization to achieve continuous adaptivity. The Q-learning component progressively learns efficient task–processor mappings through environment interaction and allows the system to respond dynamically to changing workloads or resource states without retraining. This adaptivity bridges the gap between traditional heuristics and data-driven learning-based schedulers.

GRASP integrated with Simulated Annealing has previously shown potential in homogeneous platforms^26^, improving local search yet remaining single-objective. Comparative studies involving GRASP, Tabu Search, Simulated Annealing, Genetic Algorithms, HEFT, and First Come First Served (FCFS)^20^ indicate GRASP’s competitive performance in minimizing completion time. However, these algorithms generally overlook essential objectives such as energy efficiency, fault tolerance, and reliability, which are increasingly important in large-scale heterogeneous systems.

Multi-objective extensions have also been explored. Shirvani et al.^21^ introduced a bi-objective SA algorithm for hybrid clouds optimizing makespan and cost, but without joint objective integration. Akbari et al.^22^ developed the HACG algorithm integrating Cuckoo Optimization with GA to improve resource utilization, though the weighted-sum method limited Pareto diversity. Zahra et al.^27^ applied Integer Linear Programming (ILP) for fog computing with a focus on execution time and energy but did not consider scalability or adaptability.

Further hybrid evolutionary–heuristic methods include Prashant et al.^28^, who combined fuzzy task clustering with Harmony Search and GA for fog–cloud workflows. Behera et al.^23^ merged the Grey Wolf Optimizer with GA and achieved improvements in makespan and energy at the cost of increased parameter sensitivity. Imene et al.^24^ employed NSGA-III to optimize time, cost, and energy, outperforming NSGA-II but at higher computational complexity. Despite improved convergence, these algorithms lack runtime adaptability and underperform in dynamic heterogeneous systems.

Saad et al.^29^ made notable progress by integrating MOEA/D with GRASP and Simulated Annealing, achieving strong makespan and parallelism results. However, their approach exhibits three key limitations: high computational costs for large workflows, absence of genuine adaptivity for dynamic environments, and lack of energy and reliability considerations. Similarly, recent approaches like REMO^30^ improved Pareto front regularity but remain unexplored for dynamic workflows.

The proposed QLSA-MOEA/D framework directly addresses these gaps through three major contributions. First, it employs reinforcement learning for genuine adaptivity to enable real-time adjustment to system changes without retraining. Second, it optimizes multiple conflicting objectives-makespan, energy, and reliability-beyond the limited focus of prior studies. Third, it integrates Q-learning with Simulated Annealing within MOEA/D to reduce computational complexity while maintaining solution quality. This integration overcomes the limitations identified in Hosseini’s taxonomy and earlier hybrid designs and offers a robust and efficient scheduling strategy for both static and dynamic heterogeneous computing environments.

## System model

In heterogeneous computing environments, the workflow is represented as a Directed Acyclic Graph (DAG). Each vertex $T_i \in T$ denotes a unique computational task, while each edge $e_i \in E$ specifies a precedence constraint between tasks. Tasks without predecessors form the *entry set* $T_{\text {entry}}$, whereas tasks without successors form the *exit set* $T_{\text {exit}}$. The size of the DAG equals the total number of tasks $|T|$.

Execution on a given processor assumes negligible intra-processor communication delay. However, an edge weight represents the inter-processor communication cost when tasks are executed on different units.

A task becomes *ready* once all its parent tasks have finished execution and the required data is available on its assigned processor. Ready tasks are then selected for scheduling according to the adopted allocation strategy.

**Fig. 1:**
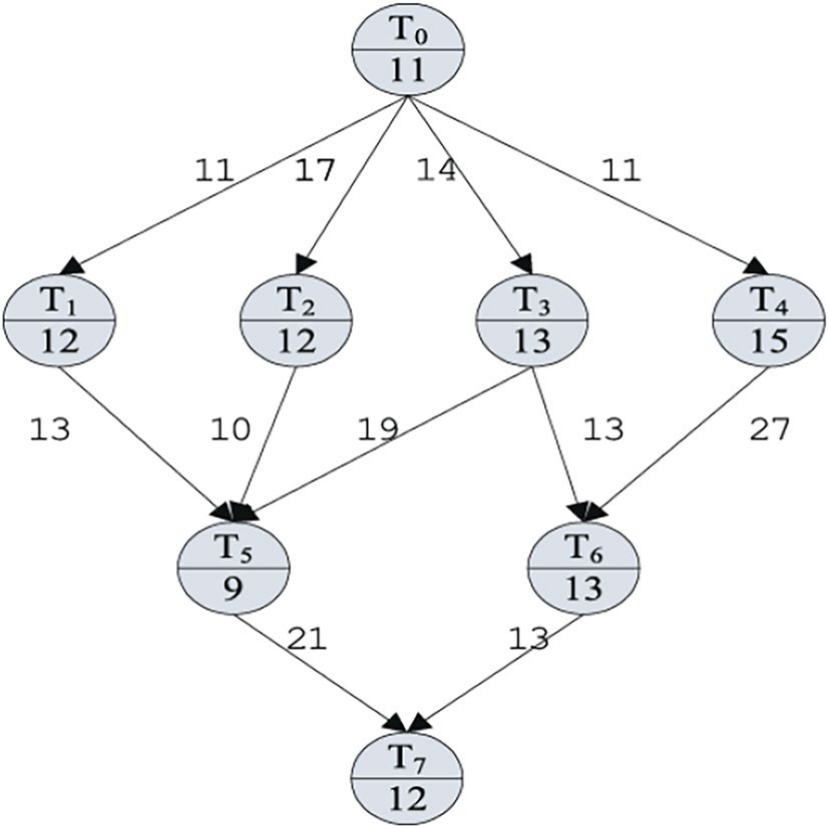
Example of a Directed Acyclic Graph (DAG)^31^.

Figure 1 illustrates a sample DAG with task dependencies. Table 3 reports example execution speeds and computation costs for multiple heterogeneous processors.

**Table 3:** Execution speed and computation cost across heterogeneous processors^31^.

$t_i$	Speed			Cost		
	$P_0$	$P_1$	$P_2$	$P_0$	$P_1$	$P_2$
$t_0$	1.00	0.85	1.22	11	13	9
$t_1$	1.20	0.80	1.09	10	15	11
$t_2$	1.33	1.00	0.86	9	12	14
$t_3$	1.18	0.81	1.30	11	16	10
$t_4$	1.00	1.37	0.79	15	11	19
$t_5$	0.75	1.00	1.79	12	9	5
$t_6$	1.30	0.93	1.00	10	14	13
$t_7$	1.09	0.80	1.20	11	15	10

### Task graph generation metrics

#### Computation cost

Each task $t_i$ has a base workload $W(t_i)$. Its execution time on a processor $P_x$ is given by Eq.(1):

1 $$\begin{aligned} W(t_i, P_x) = \frac{W(t_i)}{S(t_i, P_x)} \end{aligned}$$

where $S(t_i, P_x)$ is the processing speed of $P_x$ when executing $t_i$.

#### Communication-to-computation ratio (CCR)

The CCR measures the balance between computation time $W(t_i)$and communication delay $C(t_i, t_j)$ as defined in Eq.(2):

2 $$\begin{aligned} \text {CCR} = \frac{\frac{1}{|E|} \sum _{(t_i, t_j) \in E} C(t_i, t_j)}{\frac{1}{|N|} \sum _{t_i \in N} \overline{W(t_i)}} \end{aligned}$$

Low CCR values indicate computation-intensive workloads, while high CCR values imply communication-dominated workflows.

### Scheduling mechanism on heterogeneous processors

The *Heterogeneous Earliest Finish Time* (HEFT) algorithm^13^ determines both task order and processor allocation. It relies on the following time metrics:

- **Earliest start time (EST):**
  3 $$\begin{aligned} \text {EST}(t_i, P_x) = {\left\{ \begin{array}{ll} 0 & \text {if } t_i \in T_{\text {entry}} \\ \max \limits _{t_j \in \text {Pred}(t_i)} \left\{ \begin{aligned} & \text {AFT}(t_j), & \text {if } P_j = P_x \\ & \text {AFT}(t_j) + C(t_j, t_i), & \text {otherwise} \end{aligned} \right. \end{array}\right. } \end{aligned}$$
- **Actual start time (AST):**
  4 $$\begin{aligned} \text {AST}(t_i, P_x) = \max (\text {EST}(t_i, P_x), \text {Avail}(P_x)) \end{aligned}$$
- **Earliest compilation time (EFT):**
  5 $$\begin{aligned} \text {EFT}(t_i, P_x) = \text {AST}(t_i, P_x) + W(t_i, P_x) \end{aligned}$$
- **Actual compilation time (AFT):**
  6 $$\begin{aligned} \text {AFT}(t_i) = \min _{1 \le x \le m} \text {EFT}(t_i, P_x) \end{aligned}$$
- **Makespan:**
  7 $$\begin{aligned} \text {Makespan} = \max _{t_i \in N} \text {AFT}(t_i) \end{aligned}$$

### Optimization objectives

In heterogeneous task scheduling, the problem is formulated as a **bi-objective optimization** problem with two main goals:

1. **Minimize the makespan**, as defined in Eq. (7), which represents the total completion time of the workflow.
2. **Maximize resource utilization**, reflecting system-level parallelism and load balancing among processors^22^.

To quantify resource utilization, the **load balance efficiency** metric ($\beta$) is used. It measures how evenly the tasks are distributed across all available processors and is defined as:

8 $$\begin{aligned} \beta = \frac{1}{\sum _{i=0}^{p-1} \left| \frac{T}{p} - |p_i| \right| } \end{aligned}$$

where *T* denotes the total number of tasks in the workflow, *p* represents the number of available processors, $|p_i|$ is the number of tasks assigned to processor $p_i$, and $\frac{T}{p}$ corresponds to the ideal balanced load per processor. The denominator $\sum _{i=0}^{p-1} \left| \frac{T}{p} - |p_i| \right|$ represents the total absolute deviation from perfect load balancing among all processors. The $\beta$ metric has the following properties:

- Higher $\beta$ values indicate better load balancing and more efficient resource utilization.
- Lower $\beta$ values indicate higher workload imbalance across processors.

Therefore, the optimization process aims to **minimize makespan** while **maximizing load balance efficiency** ($\beta$) to achieve effective task scheduling in heterogeneous computing environments.

## The proposed integrated framework

This study employs the Multi-Objective Evolutionary Algorithm based on Decomposition **(MOEA/D)** as the core framework for task scheduling in heterogeneous computing environments. The optimization process is decomposed into multiple single-objective subproblems, each represented by a uniformly distributed weight vector. These subproblems are grouped into neighborhoods for information exchange. This grouping helps maintain population diversity. It also promotes the discovery of well-distributed solutions across the search space. This decomposition structure enhances both exploration capability and optimization efficiency.

To further improve MOEA/D performance, the proposed approach integrates Reinforcement Learning (RL) and metaheuristic search. The framework specifically uses Q-Learning and Simulated Annealing (SA). Within this integrated design, RL acts as a learning-based strategy, while SA provides a local search mechanism to enhance the convergence behavior of MOEA/D. Instead of a purely random initialization, the proposed QLSA strategy uses Q-Learning and SA to generate a more informed starting population and results in faster convergence toward high-quality schedules.

The primary objective of this hybrid QLSA-MOEA/D framework is to generate task schedules that minimize makespan, improve resource utilization, and ensure timely execution of all tasks. The subsequent subsections detail the proposed method: Subsection 4.1 describes the MOEA/D initialization phase, and Subsection 4.2 explains the integration of QLSA into MOEA/D.

### Initialization phase

Initialization plays a key role in influencing MOEA/D’s search behavior. Here, the population is generated using structured, learned techniques instead of relying solely on randomness. This approach enhances the algorithm’s ability to explore the Pareto front, where the best trade-offs between objectives are located.

#### Q-learning

In the initialization stage, QLSA uses reinforcement learning to build efficient task schedules without predefined rules. During training, the agent builds schedules step by step. It interacts with the environment over $E_ql$ episodes. At the start of each episode, the schedule is set to null, and the list $available_T$ contains only tasks whose dependencies have been satisfied.

At each decision point, the current state is encoded as $state \leftarrow encode(schedule)$, representing the current partial order of tasks. The agent then selects the next task $t_i$ using an $\epsilon$-greedy strategy: with probability $\epsilon$, a task is randomly chosen from $available_T$, and with probability $1 - \epsilon$, the task with the highest Q-value is selected. The chosen task is appended to the schedule, and $available_T$ is updated accordingly.

After scheduling a task, the next state becomes $nextState \leftarrow encode(schedule)$. The agent then receives a reward based on the schedule quality. $reward \leftarrow -makespan(schedule)$^7^. For the CyberShake workflow with three-objective optimization, the reward incorporates makespan, resource utilization, and energy consumption in a weighted combination. The Q-value is then updated using the temporal difference formula^12,32^:

9 $$\begin{aligned} Q(state, t_i) \leftarrow Q(state, t_i) + \alpha \left[ reward + \gamma \cdot \max _{a'} Q(nextState, a') - Q(state, t_i) \right] \end{aligned}$$

where $\alpha$ is the learning rate, $\gamma$ is the discount factor, and $a'$ denotes the available actions in the new state. The process continues until the entire schedule is constructed. After each episode, the exploration rate decays. Through repeated interaction, the agent gradually learns task–processor mappings. These mappings minimize the makespan. During deployment, the trained Q-table is employed to construct new schedules greedily using the best Q-values at each state. The generated schedules are subsequently refined through local search methods, such as Simulated Annealing, to further enhance solution quality in heterogeneous systems^6^.

An additional advantage of this framework is its adaptability to dynamic environments. When a new task arrives during execution, the scheduler dynamically updates $available_T$ to include the new task and its dependencies. The Q-learning agent, already trained to generalize scheduling decisions, integrates the new task by continuing the decision-making process based on the updated DAG state. This enables the framework to maintain scheduling stability and efficiency even under runtime variations, where new tasks or dependencies may appear.

Overall, Q-learning serves as a core mechanism for adaptive schedule generation, capable of handling both static and dynamic workflows without retraining from scratch. The training procedure is summarized in Algorithm 1.

**Algorithm 1:**
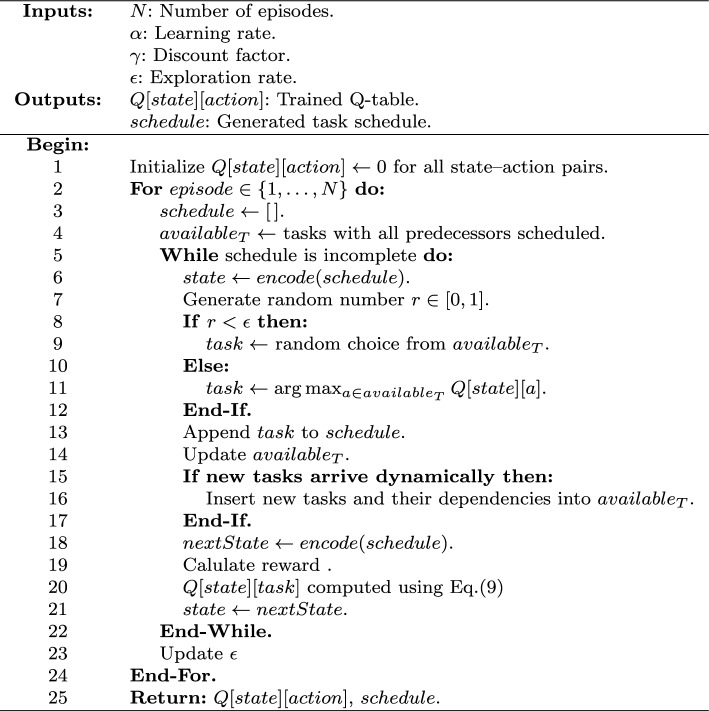
Q-Learning Training Procedure.

#### Search via simulated annealing in QLSA

Once training is complete, the learned Q-table is used to construct an initial task schedule by greedily selecting actions with the highest Q-values. The learned policy is exploited during schedule construction. However, the schedule may still be suboptimal. Limited exploration or imperfect rewards during training can cause this.

To improve the initial schedule solution, Simulated Annealing (SA) is applied. This stochastic local search technique helps escape local minima by accepting worse solutions with a controlled probability. The SA algorithm starts with an initial temperature *Temp* that influences acceptance of inferior moves^26^.

At each step, two tasks $t_i$ and $t_j$ are randomly selected and swapped to form a new candidate solution $S_1$. The acceptance decision depends on the makespan difference and is computed as:

10 $$\begin{aligned} P_{\text {Accept}} = \left\{ \begin{array}{ll} 1 & \text {if } F_{S_1} \le F_S \\ \exp \left( \frac{F_S - F_{S_1}}{Temp} \right) & \text {otherwise} \end{array} \right. \end{aligned}$$

Here, $F_S$ and $F_{S_1}$ denote the makespan of the current and new solutions, respectively. The probability of accepting worse solutions decreases as *Temp* cools down or the fitness function gap widens.

After each iteration, the temperature is updated using a decay rate *r*, and the process continues until a stopping condition is met. The best-found schedule is returned. The full QLSA procedure is shown in Algorithm 2.

**Algorithm 2:**
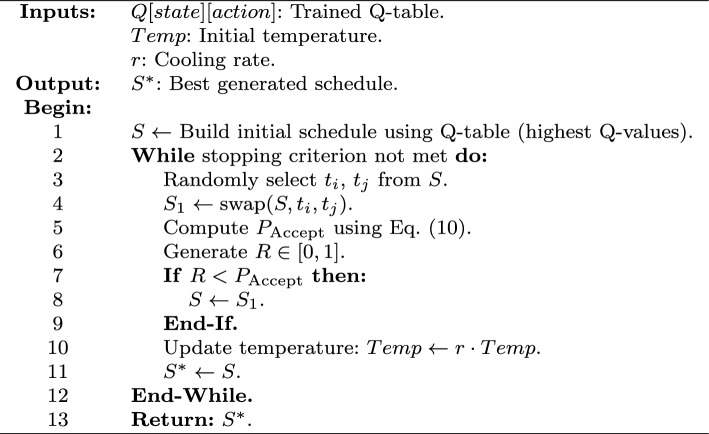
QLSA Scheduling Procedure.

This hybrid method combines reinforcement learning with probabilistic local search. As a result, QLSA generates high-quality task schedules that adapt to large and dynamic heterogeneous computing environments.

### MOEA/D

The Multi-Objective Evolutionary Algorithm based on Decomposition (MOEA/D) is a powerful framework designed to solve optimization problems with multiple conflicting objectives. Unlike traditional methods that treat all objectives together, MOEA/D breaks down a complex multi-objective problem into several simpler scalar subproblems. Each subproblem focuses on a specific combination of objectives, which simplifies the overall optimization process^33–35^.

#### Chromosome representation

**Fig. 2:**
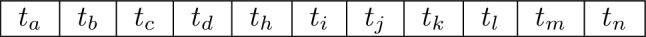
Example of a Scheduled Task Queue (STQ) in DAG scheduling.

In this framework, each chromosome encodes a candidate schedule as an ordered list of tasks. These tasks form a sequence that respects the dependencies in a Directed Acyclic Graph (DAG). The chromosome length matches the total number of tasks. Such a candidate solution is called a Scheduled Task Queue (STQ), which explicitly represents the order of task execution. Figure 2 shows an example of an STQ where $t_a$ denotes task $a$.

#### Fitness function for multi-objective scheduling

MOEA/D finds solutions that balance competing objectives. To evaluate solution quality, a weighted-sum approach is adopted to combine multiple objectives into a single scalar value suitable for comparison^29^.

The scheduling problem is formulated as a bi-objective optimization task with two primary goals: minimizing the overall makespan and maximizing resource utilization ($\beta$), corresponding to Eqs. (7) and (8). Since the MOEA/D framework inherently handles objectives in minimization form, the maximization of $\beta$ is transformed into an equivalent minimization. The combined multi-objective fitness function (MOF) is expressed as:

11 $$\begin{aligned} \text {Minimize } MOF = \lambda \times \text {Makespan} + (1 - \lambda ) \times \frac{1}{\beta } \end{aligned}$$

In the proposed framework, weight vectors $\lambda$ are predefined and uniformly distributed following MOEA/D principles. Each vector represents one subproblem to ensure diverse Pareto-front coverage^33,36^. A set of weight vectors $\lambda = [\lambda _1, \lambda _2]$ is generated before the optimization process to represent different trade-offs between objectives. Each vector corresponds to one scalar subproblem, ensuring a well-distributed approximation of the Pareto front. This mechanism provides mathematical consistency and diversity preservation without requiring manual selection of an optimal $\lambda$ value. Here, $\lambda \in [0, 1]$ controls the relative importance of each objective.

This formulation ensures that smaller makespan values and higher resource utilization levels both contribute to a lower MOF and maintains consistency within the minimization framework. Hence, the trade-off between objectives is preserved while enabling unified evaluation of candidate solutions in heterogeneous computing environments.

Unlike Shirvani et al.^21^, who optimized each objective independently, the proposed **QLSA-MOEA/D** jointly optimizes multiple conflicting goals through decomposition-based multi-objective optimization. In this framework, each subproblem represents a scalar aggregation of objectives such as makespan minimization and resource utilization maximization:

$$\begin{aligned} g_i(x) = \lambda _1 f_1(x) + \lambda _2 f_2(x), \end{aligned}$$

where $f_1(x)$ denotes the normalized makespan and $f_2(x)$ represents the normalized resource utilization factor ($\beta$). The weighting vector $\lambda = [\lambda _1, \lambda _2]$ defines the trade-off between these objectives, ensuring that the algorithm explores diverse Pareto-optimal solutions. Consequently, **QLSA–MOEA/D** performs genuine joint optimization across multiple performance metrics rather than treating them independently.

#### MOEA/D reproduction operators

1) Selection Strategy This work employs tournament selection^20,29,37^ to choose parent individuals for reproduction. A subset of individuals, sized Tourn-Size, is randomly sampled from the population. Among this subset, the two individuals with the highest fitness, denoted $S_i$ and $S_j$, are selected to produce offspring. The selection process is summarized in Algorithm 3.

**Algorithm 3:**
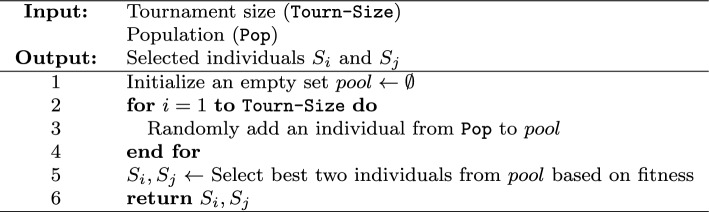
Tourn-Selection(Tourn-Size, Pop).

**Fig. 3:**
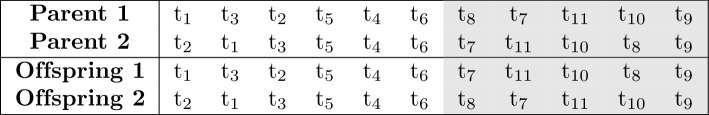
Crossover example.

**Fig. 4:**
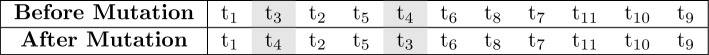
Mutation example.

2) Crossover Strategy Crossover is performed on the selected parent chromosomes obtained from the tournament selection^29,37,38^. With crossover probability $p_c$, one or more crossover points are chosen randomly. Segments between parents are swapped at these points to create offspring chromosomes. This operation promotes genetic diversity by exploring new solutions. Figure 3 illustrates the crossover process.

3) Mutation Strategy Mutation helps preserve population diversity by introducing small random changes to offspring chromosomes^29,37,38^. It is applied with a low mutation probability $p_m$. Here, mutation swaps two randomly chosen tasks within a chromosome. This technique reduces the risk of premature convergence while maintaining solution quality. Figure 4 depicts an example of the mutation operation.

The QLSA-MOEA/D in Algorithm 4 starts by creating an initial population composed of multiple candidate solutions, each representing a potential schedule. This initialization is performed by invoking the **InitializePopulation** procedure based on the QLSA scheduling approach, which generates the population $Pop$.

Once the population is formed, the algorithm evaluates the fitness of each individual, considering two main objectives: minimizing the makespan and maximizing resource utilization. These objectives are integrated into a multi-objective fitness function (MOF).

In every generation, two-parent solutions $S_i$ and $S_j$ are selected from the current population using a tournament selection method. If a randomly generated number is below the crossover probability $P_c$, the parents undergo crossover to produce offspring $S_i'$ and $S_j'$. Then, mutation is applied on these offspring with probability $P_m$, yielding the mutated offspring $S_i''$ and $S_j''$.

**Fig. 5:**
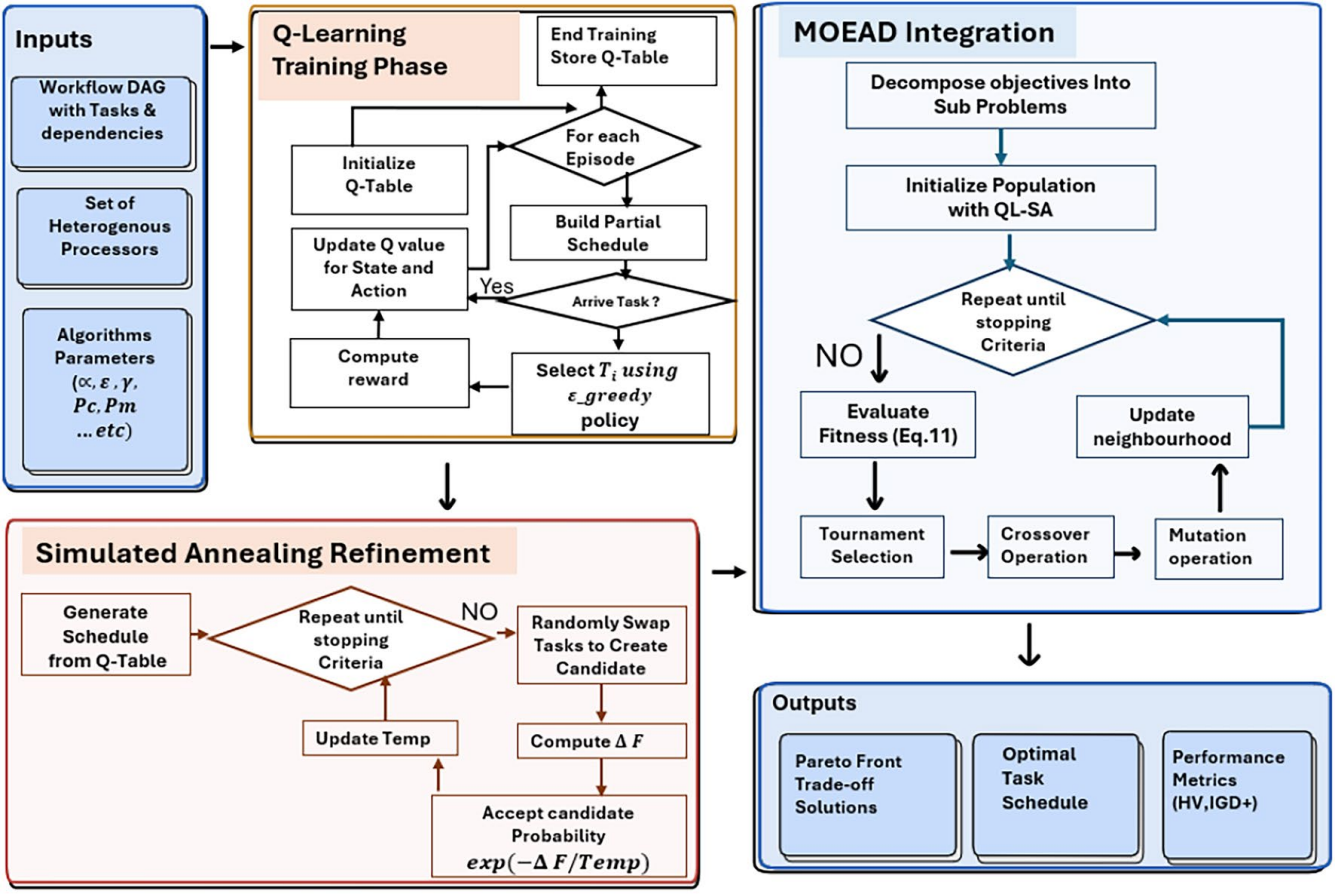
The Flowchart of QLSA-MOEAD.

The newly mutated offspring are then evaluated. Typically, one offspring $(S_i'')$ is compared with its neighboring solutions within the population. If it has better fitness, it replaces the weaker neighbor. This evolutionary process repeats until a stopping condition is fulfilled. The best solution found during this process is then returned as $S^*$. The flowchart of the QLSA-MOEAD framework is introduced in Fig. 5.

**Algorithm 4:**
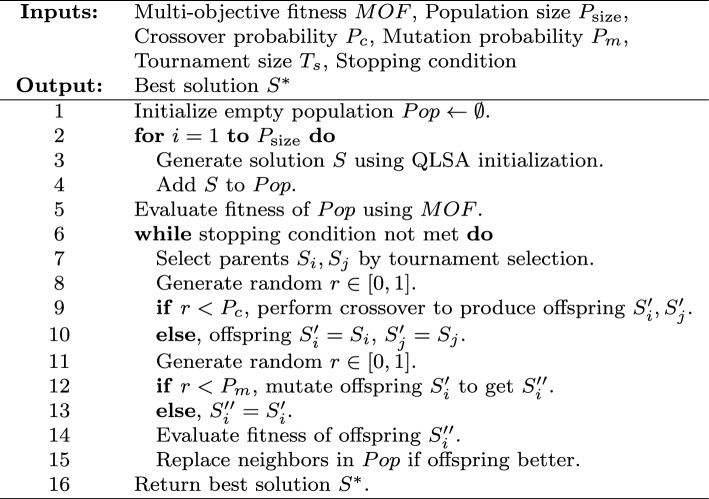
MOEA/D with QLSA Initialization.

## Performance evaluation

This section evaluates the proposed QLSA-MOEAD framework by comparing it against state-of-the-art methods: GRASP-MOEA/D, Tabu Search based MOEAD (TS-MOEA/D), Simulated Annealing based MOEAD (SA-MOEA/D), Guided Tabu Search based MOEA/D (GTS-MOEA/D), GSA-MOEA/D^29^, and HACG-TS^22^. The evaluation employs multiple performance metrics on diverse workflow benchmarks under both static and dynamic scheduling scenarios.

### Multi-objective performance metrics

- **Hypervolume (HV)** Hypervolume measures the volume of objective space dominated by a solution set relative to a reference point. Higher HV values indicate better convergence toward the Pareto front and improved solution diversity^39^. Let *HV*(*O*) denote the hypervolume of solution set *O* with reference point *U*. The metric is computed as:
  12 $$\begin{aligned} HV(O) = \sum _{o \in O} \prod _{i=1}^{m} \left( U_i - O_i \right) \end{aligned}$$
  where $O_i$ represents the *i*-th objective value of solution *o*, $U_i$ is the reference value for the *i*-th objective, and *m* denotes the total number of objectives.
- **Inverted Generational Distance Plus (IGD+)** IGD+ quantifies the average minimum Euclidean distance from each reference point $r \in R$ to the closest obtained solution $o \in O$, reflecting approximation quality to the reference Pareto front:
  13 $$\begin{aligned} IGD^+(O, R) = \frac{1}{|R|} \sum _{r \in R} \min _{o \in O} \Vert r - o \Vert \end{aligned}$$
  Lower IGD+ values indicate closer approximation to the reference front. The reference set *R* combines all non-dominated solutions obtained by compared algorithms.

### Dynamic performance metrics

Two specialized metrics assess performance under real-time task arrivals:

- Response Time **(RT)** Response Time measures the computational speed when reacting to dynamic changes. It captures the time required to regenerate a schedule after task arrival, reflecting decision-making efficiency. Lower values indicate faster adaptation. Let $A_t$ denote the task arrival time and $S_t$ the schedule generation time. Response time is defined as
  14 $$\begin{aligned} RT = S_t - A_t \end{aligned}$$
- **Performance Deviation** (**PD**_HV_) Performance Deviation quantifies the relative hypervolume difference between dynamic and static execution scenarios, evaluating solution quality maintenance under dynamic conditions:
  15 $$\begin{aligned} PD_{HV} = \frac{HV_{\text {ref}} - HV_{\text {dyn}}}{HV_{\text {ref}}} \times 100\% \end{aligned}$$
  where $HV_{\text {dyn}}$ represents dynamic execution hypervolume and $HV_{\text {ref}}$ the static reference hypervolume. Lower $PD_{HV}$ values indicate minimal quality degradation.

### Benchmark workflows

The Fast Fourier Transform workflow exhibits a symmetric, well-structured topology with 34 tasks^40^. This structured pattern enables assessment of algorithm behavior under tight task dependencies. Figure 7a illustrates the workflow structure.

The molecular workflow contains 50 tasks arranged in an irregular, asymmetric layout^41^. This unstructured pattern tests algorithm adaptability to heterogeneous computational requirements. Figure 7b shows the workflow topology.

A large-scale Montage workflow was generated based on the reference implementation^42^, containing 100 tasks and 179 dependencies distributed across eight levels. This workflow evaluates scalability and dynamic adaptation under real-time task arrivals. Figure 7c depicts the complete workflow structure.

The CyberShake seismic hazard analysis workflow models earthquake simulation through 20 tasks with 32 dependencies across 5 levels^21,43^. Tasks exhibit heterogeneous computational requirements. These range from lightweight preprocessing (80–130 units) to compute-intensive seismogram synthesis (180–260 units).

**Fig. 6:**
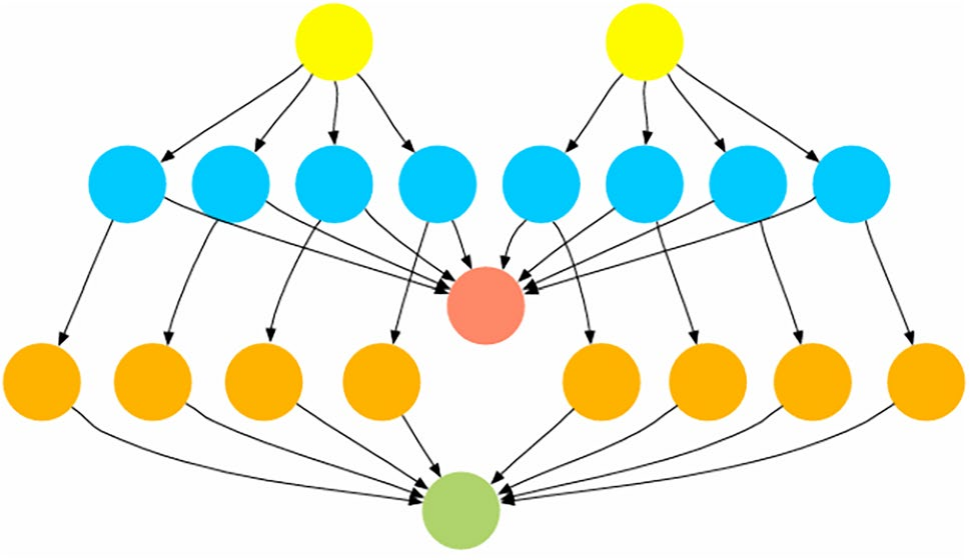
Cybershake workflow DAG for seismic hazard analysis^21,43^.

Figure 6 illustrates the workflow structure. The hierarchical pattern includes entry points, parallel processing stages, synchronization points, and final aggregation, reflecting computational requirements typical of scientific applications.

The heterogeneous evaluation platform consists of six processing units with distinct architectural characteristics, detailed in Table 4:

- **CPUs (P0, P1):** Two Intel Xeon processors (45–65W) handle control-flow operations and I/O tasks^44^.
- **GPUs (P2, P3):** Two NVIDIA units (180–250 W) accelerate data-parallel computations with a 2.5–$3.0\times$ speedup speedup^45^.
- **FPGAs (P4, P5):** Two Xilinx units (25–40W) provide energy-efficient acceleration with 1.5–$2.0\times$ speedup^46^.

Task-processor affinity is modeled: CPUs excel at I/O operations (workflow levels 0, 4), GPUs at parallel computation (levels 2, 3), and FPGAs maintain balanced performance across all levels.

Four workflow variants were created with CCR values of 0.5, 1.0, 5.0, and 10.0 following Eq. (2). These capture different scenarios: computation-intensive (CCR $< 1$), balanced (CCR $\approx 1$), and communication-intensive (CCR $> 1$). The CyberShake workflow involves three optimization objectives: makespan, resource utilization, and energy consumption.

**Table 4:** Hardware specifications for the Cybershake experimental platform.

Processor	Type	Speed Factor	Power (W)	Specialization
P0	CPU	1.0	45	Standard CPU, I/O tasks
P1	CPU	1.2	65	Fast CPU, control flow
P2	GPU	3.0	250	High-end GPU, parallel compute
P3	GPU	2.5	180	Mid-range GPU, data-parallel
P4	FPGA	1.5	25	Energy-efficient FPGA
P5	FPGA	2.0	40	Fast FPGA, pattern matching

Note: Speed factor is relative to baseline CPU (P0 = 1.0). Communication network power: 5W per active link.

### Dataset configuration

All workflows are evaluated under multiple Communication-to-Computation Ratio (CCR) values as defined in Eq. (2). CCR values above 1 indicate communication-intensive workloads, while values below 1 represent computation-intensive scenarios.

Tables 5, 6, and 7 summarize the generated datasets. Processor counts follow Amdahl’s law^40^: eight processors for FFT and molecular workflows and sixteen for Montage, ensuring fair comparison^29^.

**Fig. 7:**
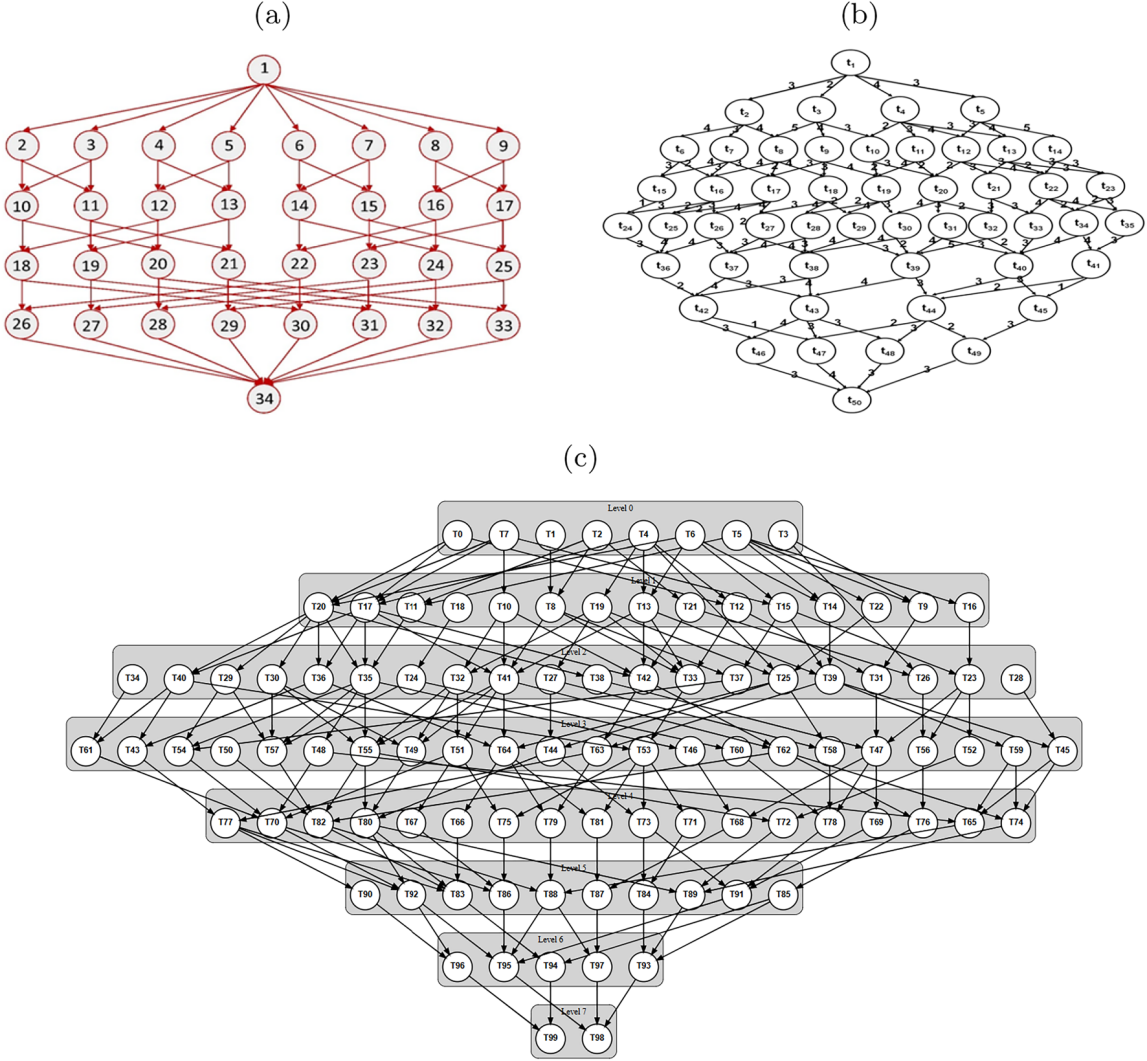
Workflow DAG topologies: **a** FFT^40^, **b** Molecular^41^, and **c** Large-scale Montage^42^.

Figures 7a , 7b , and 7c display the workflow topologies used in experiments.

**Table 5:** Generated FFT workflow datasets across multiple CCR values.

Num-Case	CCR	Num-Units	Num-Tasks	communic	comput	Type-Graph
Case 1	0.5	3	34	[5...15]	[5...30]	FFT
Case 2		8				
Case 3	1.0	3	–	[10...30]	[10...20]	–
Case 4		8				
Case 5	5.0	3	–	[50...100]	[10...20]	–
Case 6		8				
Case 7	10.0	3	–	[50...100]	[5...10]	–
Case 8		8

**Table 6:** Generated Molecular workflow datasets across multiple CCR values.

Num-Case	CCR	Num-Units	Num-Tasks	communic	comput	Type-Graph
Case 9	0.5	4	50	[5...15]	[5...30]	Molecular
Case 10		8				
Case 11	1.0	4	–	[5...15]	[5...15]	–
Case 12		8				
Case 13	5.0	4	–	[5...30]	[3...5]	–
Case 14		8				
Case 15	10.0	4	–	[10...40]	[1...5]	–
Case 16		8

**Table 7:** Generated Montage workflow datasets across multiple CCR values.

Num-Case	CCR	Num-Units	Num-Tasks	communic	comput	Type-Graph
Case 17	0.5	16	100	[2...4]	[2...8]	Montage
Case 18	1.0	–	–	[2...10]	[2...10]	–
Case 19	5.0	–	–	[20...50]	[5...10]	–
Case 20	10.0	–	–	[50...100]	[5...10]	–

Datasets are selected from established literature^21,41,47^, representing diverse communication-to-computation cost ratios for comprehensive algorithm evaluation. Sixteen static cases are designed from FFT and molecular workflows (Tables 5, 6). Four dynamic cases employ the large-scale Montage workflow (Table 7).

### Experimental setup

Experiments are run on Java (NetBeans IDE) using a 2.30 GHz processor with 15.7 GB RAM. Each test case executes 20 independent runs for statistical reliability. Average values of hypervolume (HV), inverted generational distance plus (IGD+), and response time (for dynamic scenarios) are calculated to evaluate performance. All algorithms employ the same termination criterion, which is a fixed limit of function evaluations, ensuring fair comparison across different approaches.

Parameter configurations of MOEA/D, GRASP, SA, and TS are determined from prior empirical evidence^29^. The maximum number of function evaluations is set to 1000 for all experiments to maintain consistency. Although some algorithms internally use iterations (such as simulated annealing and tabu search), the global termination condition is always defined by function evaluations for consistent computational effort across all approaches.

**Fig. 8:**
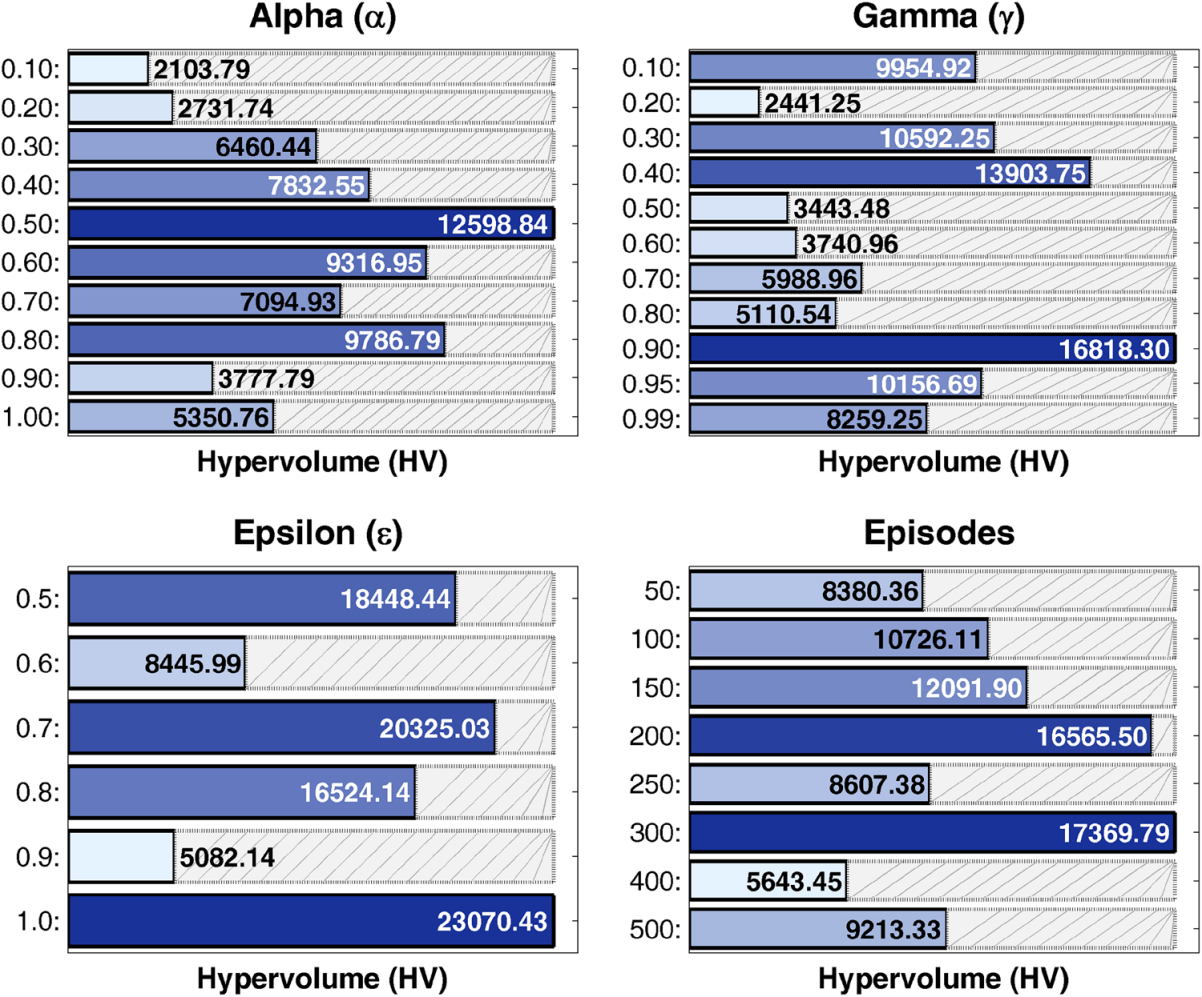
Q-learning sensitivity analysis on CyberShake workflow at CCR=1.0.

Figure 8 presents a sensitivity analysis of Q-learning hyperparameters on the CyberShake workflow at CCR=1.0. The heatmap displays hypervolume performance across learning rate $\alpha \in [0.1, 0.9]$ and discount factor $\gamma \in [0.1, 0.9]$ combinations. Several important observations emerge from this analysis. Optimal performance occurs at $\alpha =0.5$ and $\gamma =0.9$, which is the configuration used in our experiments. Performance remains stable across $\alpha \in [0.3, 0.7]$ and $\gamma \in [0.7, 0.9]$, demonstrating robustness to moderate parameter variations. Very low learning rates ($\alpha < 0.3$) slow down convergence, while very high rates ($\alpha > 0.7$) cause instability in the learning process. Low discount factors ($\gamma < 0.5$) reduce long-term planning capability, which degrades schedule quality.

Additional experiments on exploration parameters show that 300 training episodes ($E_{ql} = 300$) provide sufficient convergence for workflows up to 100 tasks. The exploration rate $\epsilon$ decays from 1.0 to 0.1 over training episodes using exponential decay ($\epsilon = \epsilon \times 0.99$ per episode) to balance exploration and exploitation effectively. Experiments with different decay rates (0.95, 0.97, and 0.99) show minimal impact on final performance, with HV variation remaining below 5%, confirming robustness to this hyperparameter.

This comprehensive analysis confirms framework robustness within reasonable parameter ranges while validating the selected configuration. Performance remains within 10% of optimal for $\alpha \in [0.3, 0.7]$ and $\gamma \in [0.7, 0.9]$. Table 8 summarizes the final configurations optimized for HV performance, applied consistently throughout the evaluation process. Configuration parameters in Table 8 are applied uniformly across all hybrid MOEA/D-based algorithms described in Section 5 to ensure fair comparison.

**Table 8:** Configuration parameters for comparative algorithms.

Algorithm	Parameter Settings
MOEA/D	Crossover rate: 70%
	Mutation rate: 30%
	Population size: 100
	Neighborhood size: 10
Tabu Search	Tabu list size: 10
	Maximum iterations without improvement: 10
Simulated Annealing	Initial temperature ($T_0$): 20
GRASP	$\alpha$ parameter: 0.4
Q-Learning	Learning rate ($\alpha$): 0.5
	Discount factor ($\gamma$): 0.9
	Exploration rate ($\epsilon$): 1.0
Maximum evaluations	1000

## Convergence and computational complexity

The workflow scheduling problem in heterogeneous environments is NP-hard, making exact convergence proofs intractable. Therefore, we provide a theoretical discussion of the convergence behavior and computational complexity of the proposed QLSA-MOEA/D framework.

### Convergence behavior

QLSA-MOEA/D integrates three complementary stochastic components: (i) Q-learning for adaptive initialization, (ii) Simulated Annealing (SA) for local refinement, and (iii) MOEA/D for evolutionary optimization.

The Q-learning component progressively improves task-processor mappings through repeated episodes, with the Q-value table converging to stable policies that effectively balance exploration and exploitation. Simultaneously, SA enhances local search capability by probabilistically accepting inferior solutions during early stages to escape local optima while gradually focusing on improvement as the temperature cools. MOEA/D decomposes the multi-objective problem into multiple scalar subproblems that evolve cooperatively through neighborhood information sharing.

Although formal global convergence guarantees remain challenging for such hybrid metaheuristics, our experimental observations demonstrate consistent monotonic improvement in both Hypervolume (HV) and Inverted Generational Distance plus (IGD+) metrics across successive generations. This empirical evidence confirms stable convergence behavior toward high-quality Pareto approximations.

### Computational complexity

Let *T* be the number of tasks, $\textit{Popsize}$ the population size, $\textit{MaxGen}$ the number of generations, $E_{\textit{ql}}$ the Q-learning episodes, and *I* the local search iterations. The overall computational complexity of QLSA-MOEA/D comprises three main components:

$$\begin{aligned} O(E_{\textit{ql}} \cdot T + I \cdot T + \textit{MaxGen} \cdot \textit{Popsize} \cdot T) \end{aligned}$$

The first term represents the Q-learning initialization cost, the second accounts for simulated annealing local search, while the third corresponds to the MOEA/D evolutionary loop. In practical scenarios, the Q-learning and SA components introduce only linear overhead relative to problem size, whereas the MOEA/D component $\textit{MaxGen} \cdot \textit{Popsize} \cdot T$ dominates the overall computational cost.

In our experimental configuration, where population size typically exceeds or equals the number of tasks ($\textit{Popsize} >= T$), the dominant term can be reasonably approximated as:

$$\begin{aligned} O(\textit{MaxGen} \cdot \textit{Popsize}^2) \end{aligned}$$

This simplification accurately reflects the observed scaling behavior and explains the final complexity representation. Table 9 presents a comparative analysis of the time complexity for QLSA-MOEA/D and other hybrid MOEA/D-based algorithms.

**Table 9:** Time complexity comparison of hybrid MOEA/D-based algorithms (dominant terms).

Algorithm	Complexity (dominant)
QLSA–MOEA/D	$O(\textit{MaxGen} \cdot \textit{Popsize}^2$
GTS–MOEA/D	$O(\textit{MaxGen} \cdot \textit{Popsize}^2)$
GSA–MOEA/D	$O(\textit{MaxGen} \cdot \textit{Popsize}^2)$
GRASP–MOEA/D	$O(\textit{MaxGen} \cdot \textit{Popsize}^2)$
TS–MOEA/D	$O(\textit{MaxGen} \cdot \textit{Popsize}^2)$
SA–MOEA/D	$O(\textit{MaxGen} \cdot \textit{Popsize}^2)$
HACG	$O(\textit{MaxGen} \cdot \textit{Popsize}^2)$

*Note:* All MOEA/D-based hybrids share the same dominant evolutionary loop complexity. QLSA-MOEA/D includes an additional linear term for Q-learning initialization, which becomes negligible for large-scale problems.

In summary, despite the incorporation of reinforcement learning for intelligent initialization, QLSA-MOEA/D maintains polynomial time complexity comparable to other hybrid variants. This favorable scaling characteristic, combined with its demonstrated solution quality advantages, ensures practical feasibility for real-world workflow scheduling applications.

## Experimental results

### Static workflows: FFT and molecular

**Fig. 9:**
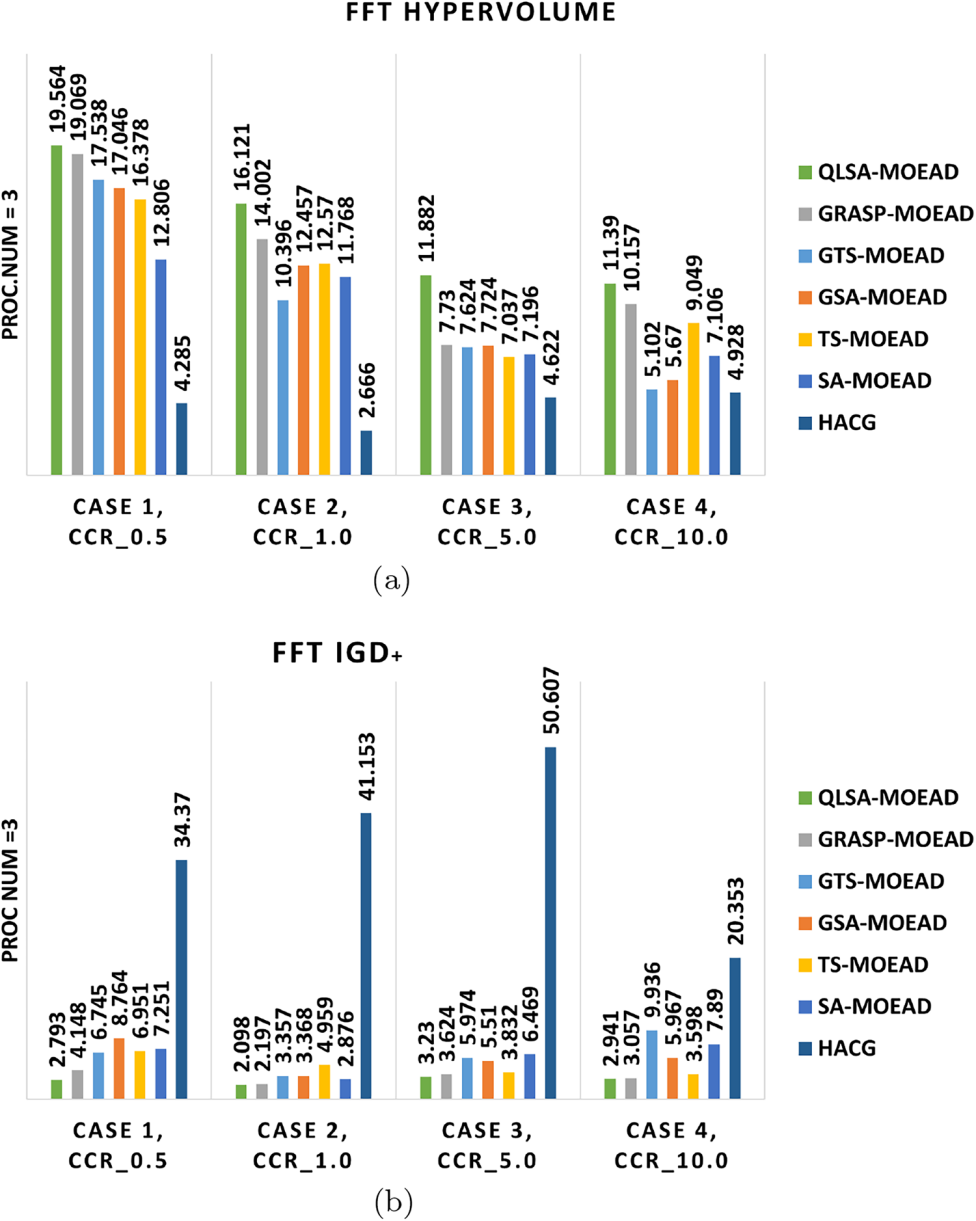
Multi-objective metrics for FFT workflow on three units under different CCR values.

**Fig. 10:**
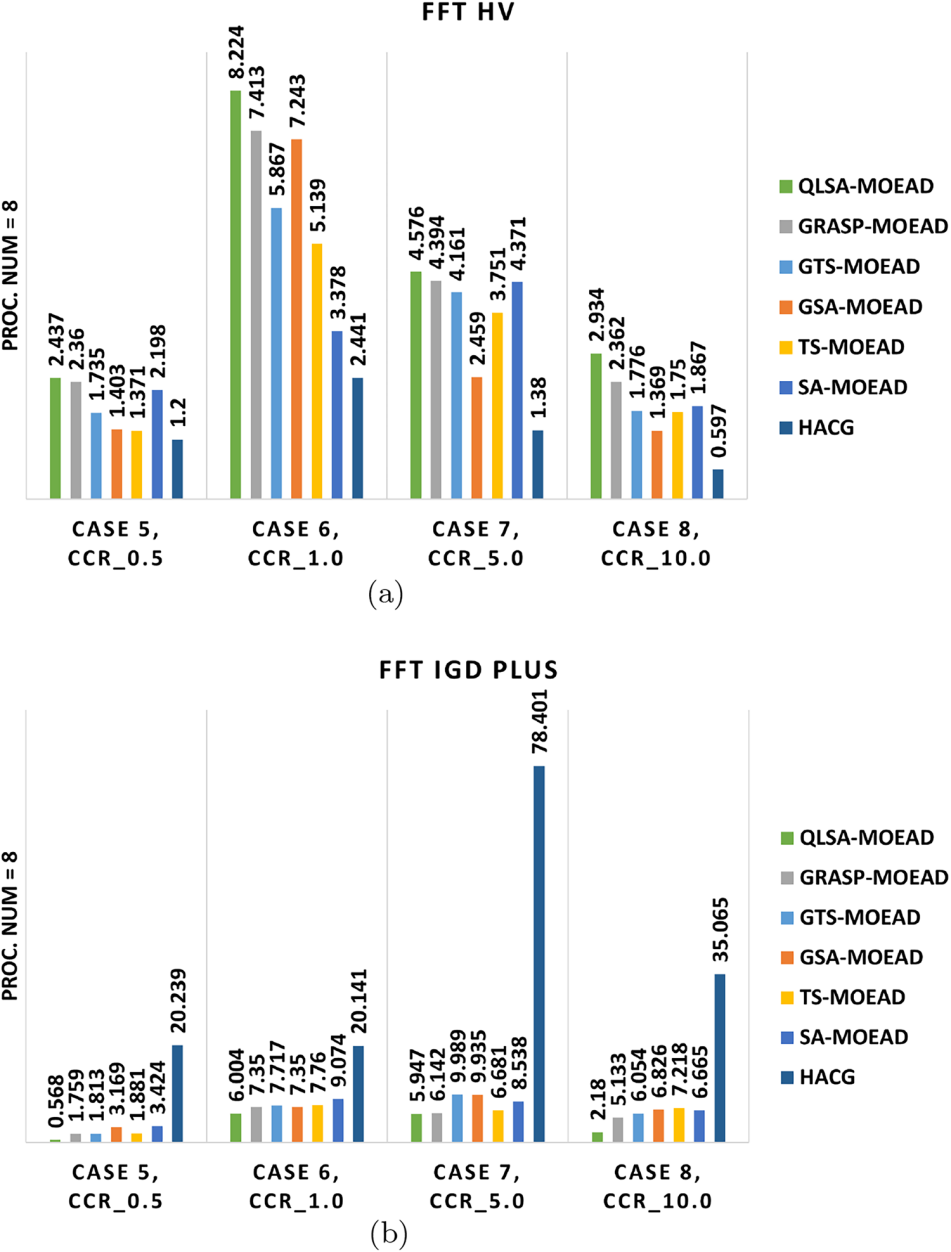
Multi-Objective Performance Indicators for FFT Workflow on Eight units.

**Fig. 11:**
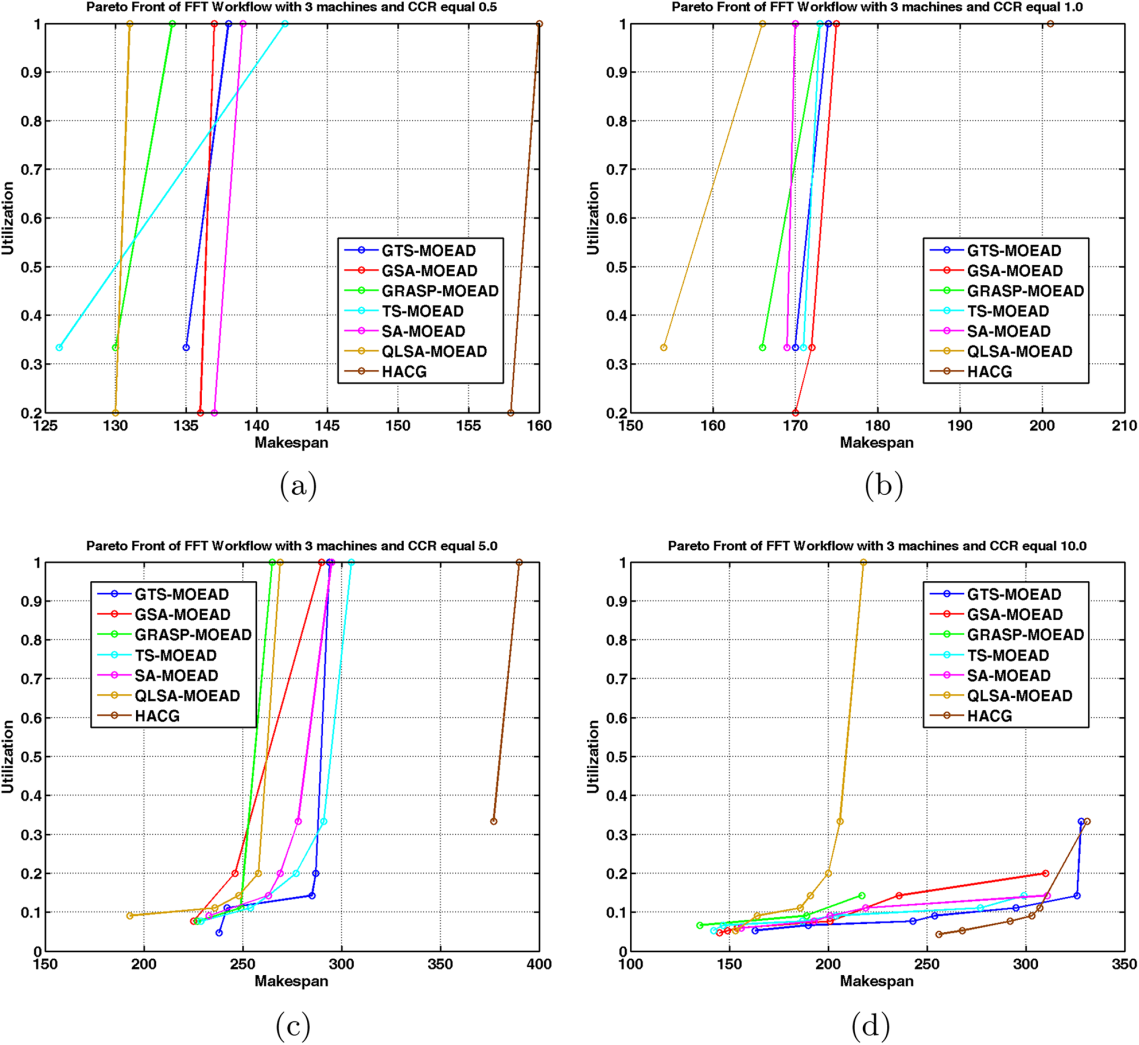
FFT Workflow Pareto Fronts under Multiple CCR with 3 units.

**Fig. 12:**
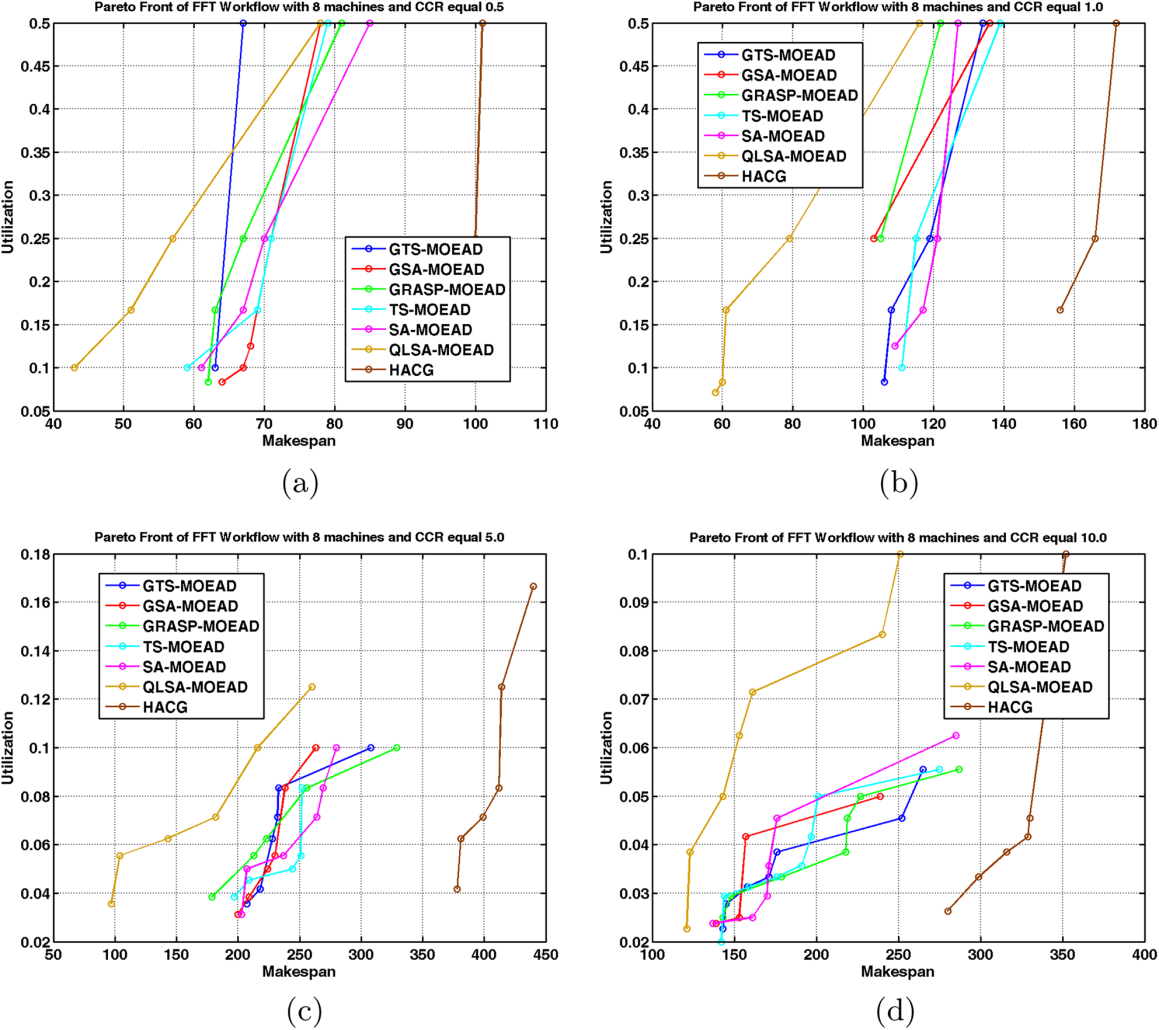
Pareto Front Distributions for FFT Workflow on Eight units.

Experiments on the structured FFT workflow used three and eight heterogeneous processors at CCR values of 0.5, 1, 5, and 10. Table 10 summarizes average hypervolume (HV) and IGD+ results. Figures 9a –10b show performance trends, while Figs. 11–12 display Pareto-front approximations.

Table 10 and Figs. 9a –10b show that QLSA-MOEAD consistently achieves the best results across both processor configurations at low CCR (0.5). The Q-learning component helps the algorithm converge faster even when task dependencies are tight, which is typical in FFT structures. GRASP-MOEAD obtains comparable HV values but shows higher IGD+. This indicates premature convergence and difficulty in maintaining diversity. GSA-MOEAD and GTS-MOEAD perform moderately well. Their local refinements improve exploitation but lack the adaptive feedback that Q-learning provides.

The performance gap widens as CCR increases to 1.0 and 5.0. QLSA-MOEAD adapts task ordering dynamically as communication overhead grows. This helps maintain stable convergence. GRASP-MOEAD and GTS-MOEAD converge slower because they rely on static initialization. They also have limited ability to rebalance loads dynamically. SA-MOEAD and TS-MOEAD show minimal improvement. Simple stochastic refinements are not enough to compensate for the structural rigidity in FFT workflows. HACG performs the weakest and demonstrates poor scalability. Diversity also decreases as dependencies increase.

At the highest CCR (10.0), QLSA-MOEAD maintains its advantage even when communication costs outweigh computation. This demonstrates a good balance between exploitation and adaptive learning. GRASP-MOEAD produces reasonable results but cannot match QLSA-MOEAD’s adaptability. Other methods like GSA-MOEAD and TS-MOEAD degrade significantly when handling communication-intensive dependencies. HACG continues producing sparse, low-quality solutions throughout.

Figures 11 and 12 confirm these observations visually. QLSA-MOEAD produces dense, well-distributed Pareto fronts, while other methods show scattered or partially converged fronts. Overall, integrating Q-learning with MOEA/D provides superior adaptability. QLSA-MOEAD works most effectively for structured FFT workflows, particularly when communication overheads are high.

The **Statistical validation** in

Table 12 presents Wilcoxon signed-rank test results comparing QLSA-MOEAD against competitors. For FFT with 3 processors, QLSA-MOEAD significantly outperforms all competitors at $p < 0.05$ across most CCR values. Only two exceptions appear: SA-MOEAD at CCR=1.0 ($p=0.1$) and TS-MOEAD at CCR=5.0 ($p=0.05$), where differences approach but do not reach strict significance. These statistical tests confirm that observed performance improvements are not random variation.

**Table 10:** AVG.Hypervolume and AVG.IGD+ results for FFT workflow executed on different heterogeneous units.

FFT	CCR	units	QLSA-MOEAD	GTS-MOEAD	GSA-MOEAD	GRASP-MOEAD	TS-MOEAD	SA-MOEAD	HACG
**AVG.HV**									
Case 1	0.5	3	**19.564**	17.538	17.046	19.069	16.378	12.806	4.285
Case 2	1.0	3	**16.121**	10.396	12.457	14.002	12.570	11.768	2.666
Case 3	5.0	3	**11.882**	7.624	7.724	7.730	7.037	7.196	4.622
Case 4	10.0	3	**11.390**	5.102	5.670	10.157	9.049	7.106	4.928
Case 5	0.5	8	**2.437**	1.735	1.403	2.360	1.371	2.198	1.200
Case 6	1.0	8	**8.224**	5.867	7.243	7.413	5.139	3.378	2.441
Case 7	5.0	8	**4.576**	4.161	2.459	4.394	3.751	4.371	1.380
Case 8	10.0	8	**2.934**	1.776	1.369	2.362	1.750	1.867	0.597
**AVG. IGD+**									
Case 1	0.5	3	**2.793**	6.745	8.764	4.148	6.951	7.251	34.370
Case 2	1.0	3	**2.098**	3.357	3.368	2.197	4.959	2.876	41.153
Case 3	5.0	3	**3.230**	5.974	5.510	3.624	3.832	6.469	111.607
Case 4	10.0	3	**2.941**	9.936	5.967	3.057	3.598	7.890	75.353
Case 5	0.5	8	**0.568**	1.813	3.169	1.759	1.881	3.424	20.239
Case 6	1.0	8	**6.004**	7.717	7.350	7.350	7.760	9.074	20.141
Case 7	5.0	8	**5.947**	9.989	9.935	6.142	6.681	8.538	78.401
Case 8	10.0	8	**2.180**	6.054	6.826	5.133	7.218	6.665	35.065

**Fig. 13:**
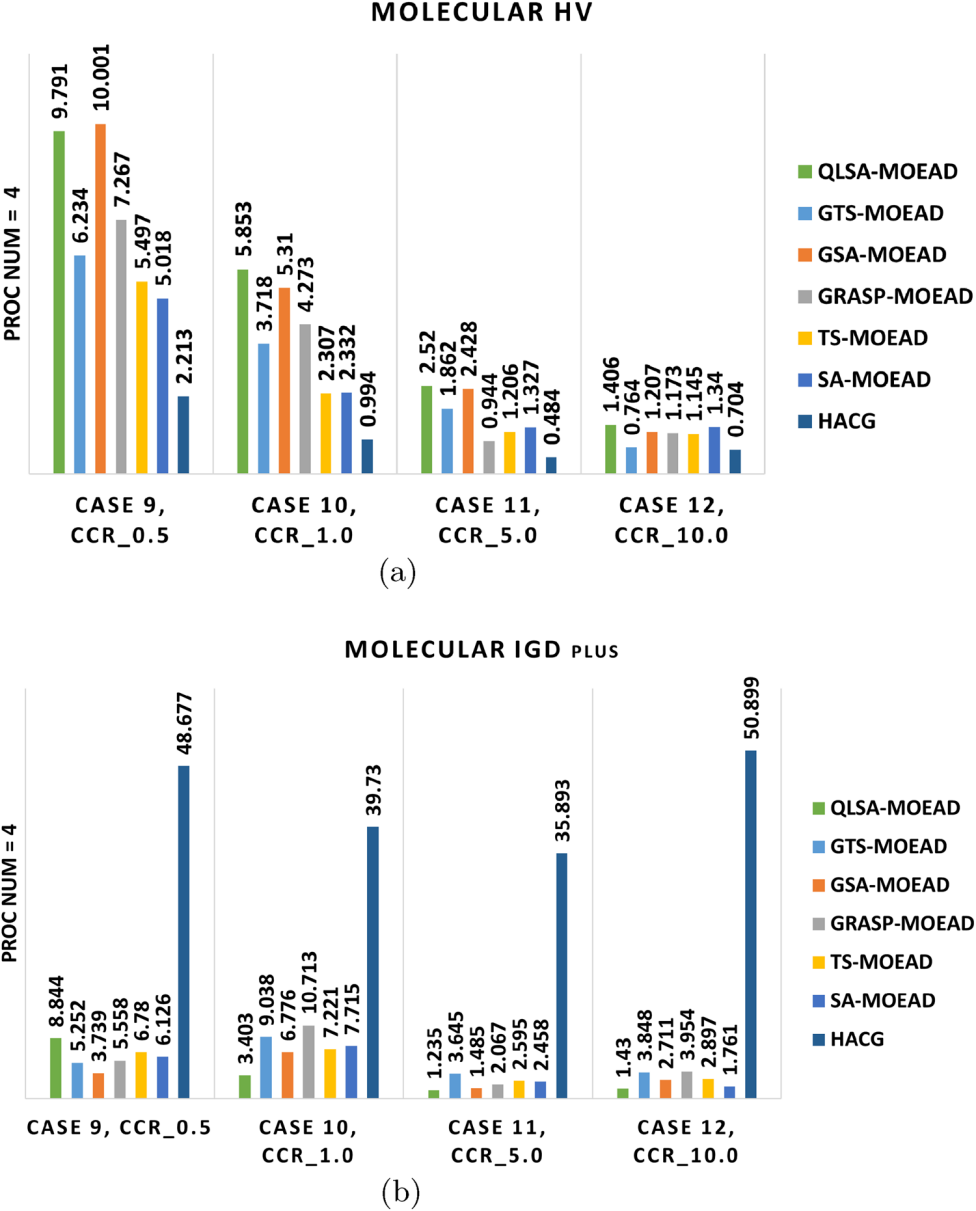
Performance metrics for multi-objective optimization of the FFT workflow using four units.

**Fig. 14:**
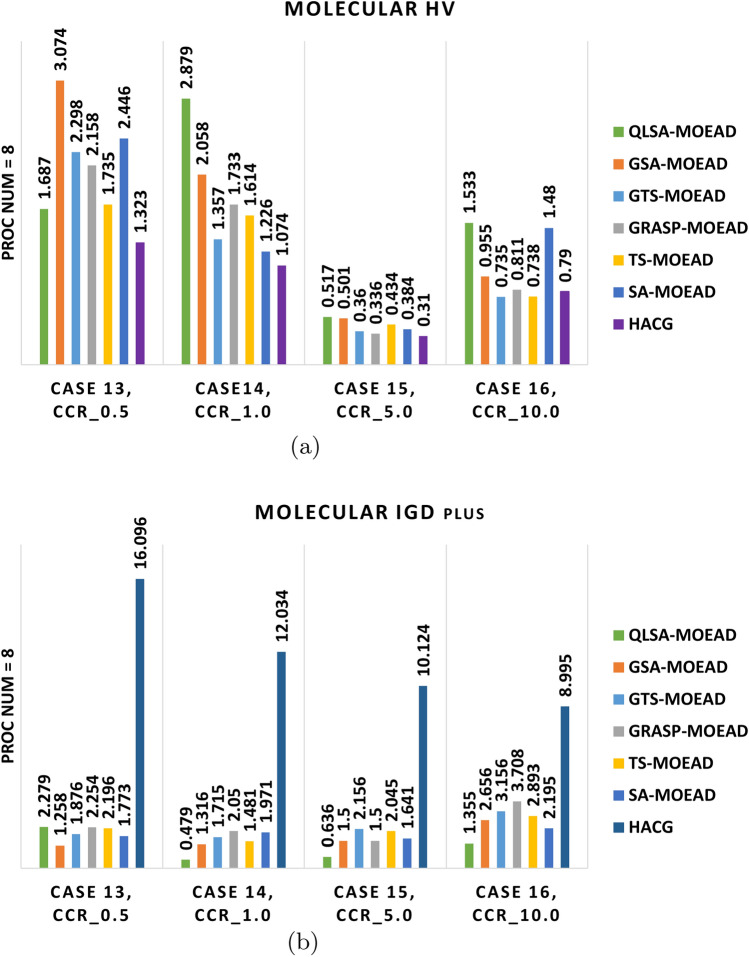
Multi-objective performance metrics for the molecular workflow on eight units.

**Fig. 15:**
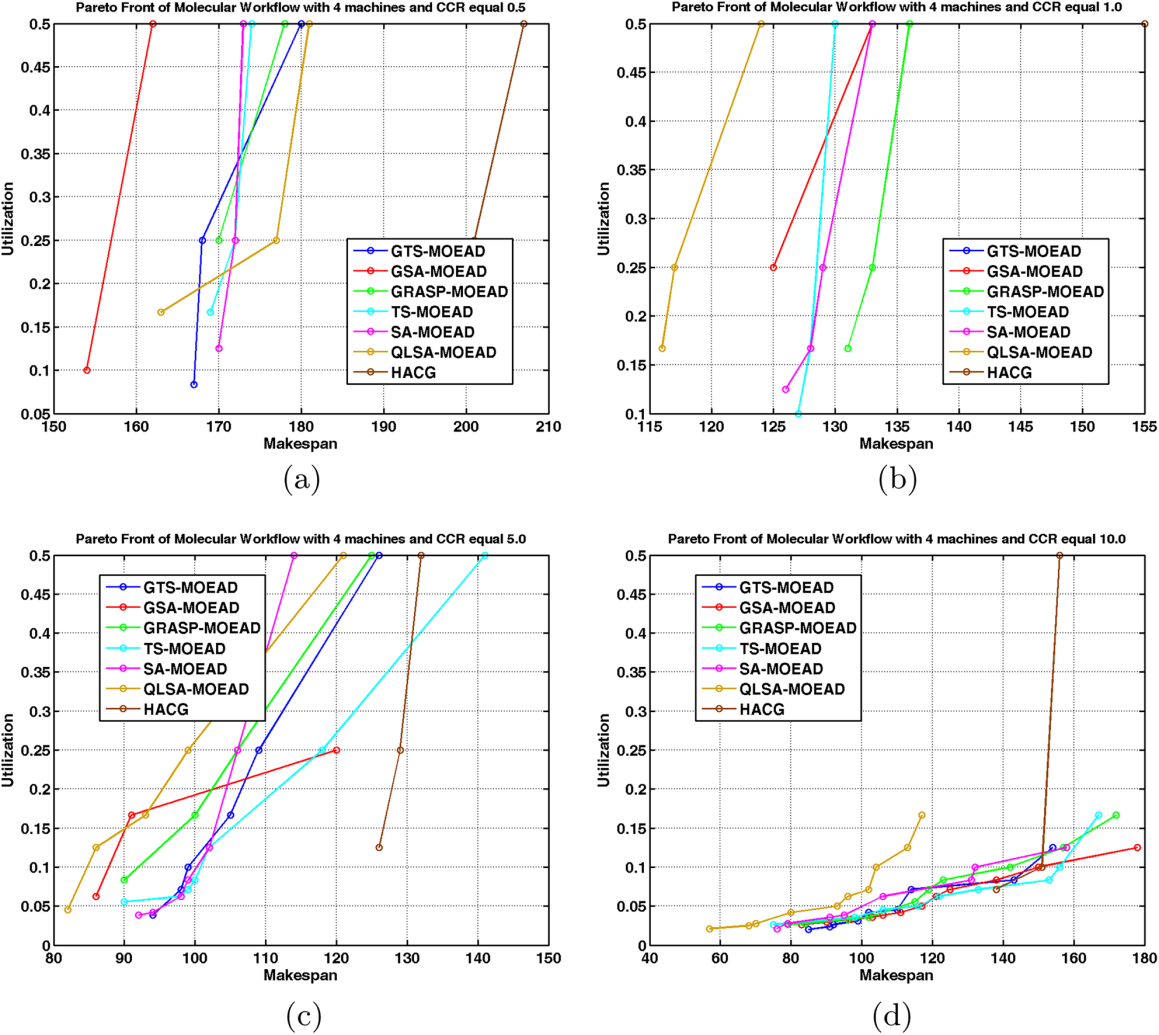
Pareto fronts of the molecular workflow on four units.

**Fig. 16:**
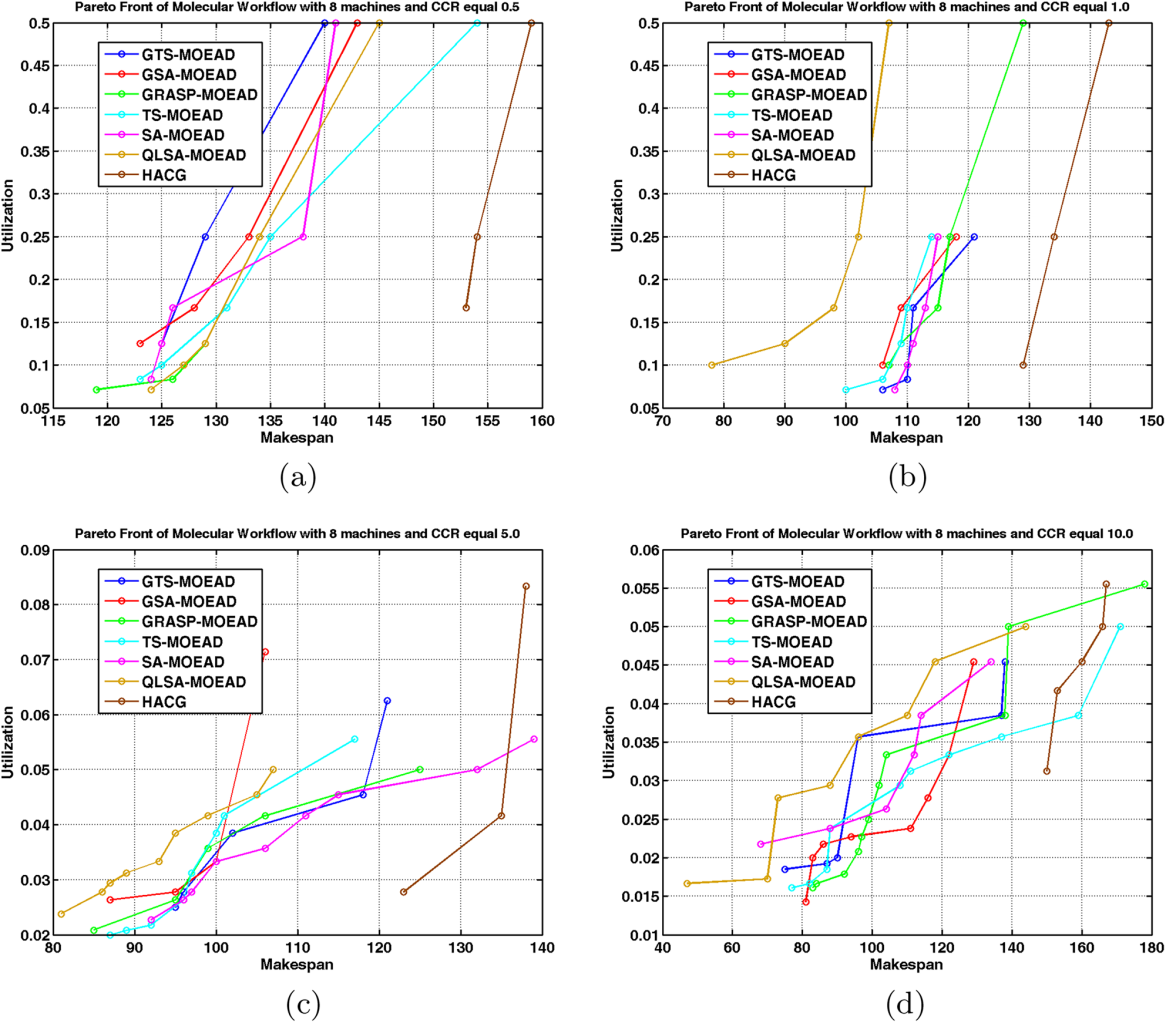
Pareto front of Molecular Workflow with different CCR and 8 machines.

We evaluated the same algorithms on the unstructured molecular workflow to assess generalizability. Experiments used four and eight processors at CCR values from 0.5 to 10. Table 11 reports HV and IGD+ values. Figures 13a –14b show performance comparisons. Figures 15 –16 displays Pareto-optimal solution distributions.

Table 11 and Figs. 13a –14b shows that the molecular workflow reveals interesting differences from FFT behavior. At low CCR with 4 and 8 processors (Cases 9 and 13), GSA-MOEAD achieves the highest HV with the lowest IGD+. GSA-MOEAD prioritizes exploration in unstructured workflows, whereas QLSA-MOEAD balances exploration and exploitation more effectively for convergence. GRASP-MOEAD, GTS-MOEAD, and TS-MOEAD show moderate performance. This reflects limited adaptability. HACG underperforms significantly throughout.

QLSA-MOEAD shows increased dominance as CCR rises to intermediate levels (Cases 10 and 14). At CCR=1.0 with 4 processors, it outperforms both GSA-MOEAD and GRASP-MOEAD in both metrics. GSA-MOEAD remains competitive but shows slower convergence under communication-intensive conditions. With 8 processors, QLSA-MOEAD substantially beats all baselines.

At high CCR values (Cases 11-12, 15-16), QLSA-MOEAD dominates. It handles high communication-to-computation ratios while preserving diversity. GSA-MOEAD retains relatively strong performance but lags slightly in convergence. SA-MOEAD occasionally approaches QLSA-MOEAD in HV but shows less uniform solution distribution based on higher IGD+ values. At 8 processors under high CCR, QLSA-MOEAD continues to excel across all test cases.

Figures 15 and 16 show QLSA-MOEAD solutions closely approximate the Pareto front. Both QLSA-MOEAD and GSA-MOEAD demonstrate consistent superiority across CCR levels and processor counts. Statistical tests in Table 12 for Molecular with 8 processors show QLSA-MOEAD significantly outperforms competitors ($p < 0.05$). The few non-significant cases appear with GRASP-MOEAD at specific CCR values and occasionally with TS-MOEAD.

Beyond solution quality, we analyzed computational efficiency to assess practical applicability. Table 13 compares execution time and HV across both workflows at CCR=1.0. Each value averages twenty independent runs. QLSA-MOEAD outperforms competitors in both speed and quality. The parallel implementation reduces computational burden without compromising convergence. Q-learning accelerates task selection through learned policies. SA refines schedules through adaptive exploration.

For the FFT workflow, QLSA-MOEAD requires substantially less time than competitors while maintaining superior HV values. The molecular workflow shows similar patterns across both processor configurations. QLSA-MOEAD provides a stable trade-off between search diversity and runtime. Lower execution time shows that reinforcement-guided initialization can replace random or greedy heuristics efficiently when supported by parallel processing. Higher HV values confirm strong convergence toward the Pareto front. The algorithm preserves solution diversity.

Compared to other hybrid methods like GSA-MOEAD and GTS-MOEAD, the proposed approach shows faster convergence and better scalability as task and processor counts increase. Traditional heuristics like GRASP, TS, and SA achieve reasonable results but lack adaptability in complex scenarios. Overall, QLSA-MOEAD effectively integrates learning with metaheuristic exploration to achieve high-quality solutions in a reasonable time.

The experimental evidence validates the algorithm’s robustness for real-world heterogeneous systems requiring both accuracy and scalability.

**Table 11:** AVG. Hypervolume and IGD+ values for the Molecular workflow on diferrent heterogeneous units.

Molecular	CCR	units	QLSA-MOEAD	GTS-MOEAD	GSA-MOEAD	GRASP-MOEAD	TS-MOEAD	SA-MOEAD	HACG
**AVG. HV**									
Case 9	0.5	4	9.791	6.234	**10.001**	7.267	5.497	5.018	2.213
Case 10	1.0	4	**5.853**	3.718	5.310	4.273	2.307	2.332	0.994
Case 11	5.0	4	**2.52**	1.862	2.428	0.944	1.206	1.327	0.484
Case 12	10.0	4	**1.406**	0.764	1.207	1.173	1.145	1.340	0.704
Case 13	0.5	8	1.687	2.298	**3.074**	2.158	1.735	2.446	1.323
Case 14	1.0	8	**2.879**	1.357	2.058	1.733	1.614	1.226	1.074
Case 15	5.0	8	**0.517**	0.36	0.501	0.336	0.434	0.384	0.31
Case 16	10.0	8	**1.533**	0.735	0.955	0.811	0.738	1.48	0.79
**AVG. IGD+**									
Case 9	0.5	4	8.844	5.252	**3.739**	5.558	6.780	6.126	48.677
Case 10	1.0	4	**3.403**	9.038	6.776	10.713	7.221	7.715	39.730
Case 11	5.0	4	**1.235**	3.645	1.485	2.067	2.595	2.458	35.893
Case 12	10.0	4	**1.43**	3.848	2.711	3.954	2.897	1.761	50.899
Case 13	0.5	4	2.279	1.876	** 1.258**	2.254	2.196	1.773	16.096
Case 14	1.0	4	**0.479**	1.715	1.316	2.05	1.481	1.971	12.034
Case 15	5.0	4	**0.636**	2.156	1.508	1.5	2.045	1.641	10.124
Case 16	10.0	4	**1.355**	3.156	2.656	3.708	2.195	2.893	8.995

**Table 12:** Wilcoxon signed-rank test of FFT with 3 processors and Molecular with 8 processors: QLSA-MOEAD vs baselines (Hypervolume). * indicates p $< 0.05$.

**Baseline**	Workflow	Processor num.	**CCR 0.5**	**CCR 1.0**	**CCR 5.0**	**CCR 10.0**
GTS-MOEAD	FFT	3	0.030*	0.0001*	0.000*	0.0001 *
GSA-MOEAD	-	-	0.02*	0.002*	0.001*	0.0002*
GRASP-MOEAD	-	-	0.01*	0.01*	0.002*	0.0001*
TS-MOEAD	-	-	0.01*	0.01*	0.05	0.0001*
SA-MOEAD	-	-	0.02*	0.1	0.000*	0.0001*
HACG	-	-	0.001*	0.01*	0.01*	0.001*
GTS-MOEAD	Molecular	8	0.04*	0.0006*	0.04*	0.03 *
GSA-MOEAD	-	-	0.02*	0.004*	0.01*	0.02*
GRASP-MOEAD	-	-	0.06	0.001*	0.08	0.04*
TS-MOEAD	-	-	0.01*	0.001*	0.02	0.04*
SA-MOEAD	-	-	0.02*	0.0001	0.04*	0.007*
HACG	-	-	0.01*	0.008*	0.0002*	0.002*

All test : QLSA-MOEAD vs baseline (one-tailed, $\alpha = 0.05$).

**Table 13:** Execution time (ms) and HV for all hybrid algorithms with MOEAD across the two workflows. Italics values represent the fastest execution time and best HV of solution per case.

Workflow	CCR	Units	QLSA	GTS	GSA	GRASP	TS	SA	HACG
**Execution Time (ms)**									
FFT	1.0	3	*4963*	13293	15949	7202	17412	15503	12630
-	1.0	8	*11686*	356638	165784	27747	352573	165099	36841
Molecular	1.0	4	*10806*	59747	24758	24147	43231	43513	64757
-	1.0	8	*72735*	120241	166909	112348	123298	123266	116141
**HV**									
FFT	1.0	3	*16.121*	10.396	12.475	14.002	12.570	11.768	2.666
-	1.0	8	*8.224*	5.867	7.243	7.413	5.139	3.378	2.441
Molecular	1.0	4	*5.853*	3.718	5.310	4.273	2.307	2.332	0.994
-	1.0	8	*2.879*	1.357	2.058	1.733	1.614	1.226	1.074

### Dynamic workflow: montage

Table 14 evaluates QLSA-MOEAD on the Montage workflow (100 tasks, 179 dependencies) under static and dynamic conditions. In static mode, average HV increases from 0.0665 at CCR=0.5 to 0.6413 at CCR=10.0, with runtime between 1,589–2,916 ms. These results establish baseline quality when schedules are computed once without disruptions.

Dynamic execution simulates real-time task arrivals requiring on-the-fly schedule adjustments. Response time (RT) remains between 0.80–1.70 ms across all CCR values, fast enough for practical deployment. The best case occurs at CCR=5.0 (RT=0.80 ms), where communication delays provide natural gaps for inserting new tasks without disrupting ongoing work.

Quality degradation varies significantly with CCR. At CCR=0.5, dynamic HV drops from 0.0665 to 0.0305 (54.1% loss) because computation-intensive workloads leave little slack time for adjustments. At CCR=1.0, degradation improves slightly to 50.6%. The most favorable scenario appears at CCR=5.0, where HV degrades only 3.7% (from 0.1547 to 0.1489). Here, communication-dominated execution creates frequent idle periods that accommodate new tasks smoothly. At CCR=10.0, degradation reaches 63.78% as extreme communication costs fragment schedules, limiting insertion flexibility.

Total runtime increases from 1,589–2,916 ms (static) to 15,356–16,207 ms (dynamic), reflecting the cumulative cost of adjustments. IGD+ values also increase from 0.4597–3.0690 (static) to 0.6713–5.3517 (dynamic), showing reduced convergence precision when schedules must adapt incrementally rather than through global optimization.

The Q-learning component enables this fast adaptation. When tasks arrive, the Q-table suggests processor assignments in constant time, SA refines the placement through local search, and MOEA/D updates only affected subproblems. This incremental process achieves 1,000–3,000$\times$ speedup over regenerating entire schedules, which would require 2,000–3,000 ms per adjustment.

In summary, QLSA-MOEAD handles dynamic scenarios with sub-millisecond response times suitable for real-time systems, though quality loss of 3.7–63.78% may not satisfy applications requiring guaranteed performance. The approach works well for cloud batch processing but less so for hard real-time control. The minimal degradation at CCR=5.0 suggests that practitioners can tune system parameters to target similar conditions and maintain near-optimal quality despite runtime changes.

**Table 14:** Static and Dynamic Montage Workflow using QLSA-MOEAD with multiple performance metrics.

Workflow	CCR	Units	HV	IGD+	RunTime (ms)	RT (ms)	$PD_{HV}$
**Static Montage Workflow**							
Montage	0.5	16	0.0665	0.7592	1589	-	-
-	1.0	16	0.1000	0.4597	2047	-	-
-	5.0	16	0.1547	3.0690	2092	-	-
-	10.0	16	0.6413	0.9966	2916	-	-
**Dynamic Montage Workflow**							
Montage	0.5	16	0.0305	2.3051	15356	1.70	54.135
-	1.0	16	0.0494	0.6713	16133	1.25	50.6
-	5.0	16	0.1489	5.3517	16207	0.80	3.7
-	10.0	16	0.2322	1.9807	15559	1.50	63.78

### Real-world validation: cyberShake workflow

The benchmark experiments on FFT, Molecular, and Montage workflows establish algorithmic effectiveness on synthetic benchmarks with controlled characteristics. The CyberShake seismic hazard analysis workflow provides validation on a realistic scientific application featuring irregular task dependencies and heterogeneous computational requirements typical of real-world scenarios.

#### Energy consumption model

The framework extends to three objectives for CyberShake: makespan, resource utilization, and energy consumption. Energy is computed using Eq. 16.

16 $$\begin{aligned} \text {Energy} = \sum _{x=1}^{P} \sum _{t_i \in P_x} \left( \text {Pow}_x \times W(t_i, P_x) \right) + \sum _{(t_i,t_j) \in E_d} \left( \text {Pow}_{\text {comm}} \times C(t_i, t_j) \right) \end{aligned}$$

where $\text {Pow}_x$ denotes the power consumption of processor $P_x$ as listed in Table 4 (Section 5.3), $W(t_i, P_x)$ represents the execution time of task $t_i$ on processor $P_x$ as defined in Eq. (1), $\text {Pow}_{\text {comm}}$ is the communication network power (5W per active link), and $C(t_i, t_j)$ denotes the communication time between tasks $t_i$ and $t_j$ for edges $(t_i, t_j) \in E_d$. The model accounts for both computational energy (processor-dependent) and data transfer energy (communication-dependent).

#### Three-objective optimization results

For the CyberShake workflow, the framework extends to three-objective optimization by incorporating energy consumption alongside makespan and resource utilization. Energy consumption is computed using Eq. 16, which accounts for both processor-specific power consumption (Table 4) and communication network power (5W per active link).

QLSA-MOEAD is evaluated against six baseline configurations: MOEAD (random initialization), SA (simulated annealing only), QL (Q-learning only), QLSA (Q-learning + SA without MOEAD), DRL^48^, and GRASP-MOEAD (current state-of-the-art hybrid)^29^. This comparison isolates component contributions while benchmarking against the best existing hybrid method.

All algorithms optimize three objectives simultaneously: makespan minimization, resource utilization maximization ($\beta$), and energy consumption minimization. For fair comparison, all multi-objective variants employ 100 uniformly distributed weight vectors following MOEAD decomposition principles. Both QL and DRL use multi-objective reward design rather than single-metric optimization, ensuring consistent learning objectives. Each algorithm executes 20 independent runs on four CyberShake variants (CCR = 0.5, 1.0, 5.0, 10.0). Table 15 presents average Hypervolume (HV) and Inverted Generational Distance Plus (IGD+).

**Table 15:** Performance comparison on CyberShake workflow across six heterogeneous units and multiple CCR values.

Case	CCR	QLSA- MOEAD	GRASP- MOEAD	QLSA	SA	QL	DRL	MOEAD
**AVG. (HV) **$\uparrow$ ($\times 10^3$)								
Case 1	0.5	**3847.41**	899.62	12.77	260.05	11.36	19.41	463.81
Case 2	1.0	**2685.37**	852.62	24.81	84.98	1.21	1.63	42.28
Case 3	5.0	**3063.90**	1332.04	26.55	265.16	11.27	3.99	56.13
Case 4	10.0	**7796.00**	3226.01	29.88	2536.32	23.58	51.09	3054.78
**AVG. (IGD+) **$\downarrow$								
Case 1	0.5	**0.2667**	0.2784	0.3117	0.2698	0.3013	0.2669	0.3201
Case 2	1.0	**0.2423**	0.2573	0.248	0.2432	0.2951	0.2973	0.2852
Case 3	5.0	** 0.2428**	0.2628	0.2914	0.2472	0.2735	0.2911	0.2788
Case 4	10.0	**0.2919**	0.3124	0.3122	0.3109	0.3203	0.3115	0.3138

HV values scaled by $10^3$ for readability. Best values per CCR in **bold**.

Table 15 demonstrates QLSA-MOEAD’s dominance across all CyberShake configurations. At CCR=0.5, the framework achieves HV of 3847.41$\times 10^3$, substantially exceeding GRASP-MOEAD (899.62$\times 10^3$) and all other baselines. This represents a 4.3-fold improvement over the previous state-of-the-art. The advantage persists across varying CCR values: at CCR=1.0, QLSA-MOEAD reaches 2685.37$\times 10^3$ versus GRASP-MOEAD’s 852.62$\times 10^3$ (3.1-fold improvement); at CCR=5.0, the gap is 3063.90 versus 1332.04 (2.3-fold); and at CCR=10.0, performance peaks at 7796.00 versus 3226.01 (2.4-fold). The IGD+ metric confirms superior convergence quality, with QLSA-MOEAD maintaining the lowest values across all cases (0.2423-0.2919).

Comparison with the DRL baseline reveals that pure deep reinforcement learning achieves only 19.41–51.09$\times 10^3$ HV, representing a 50–200-fold performance gap. This confirms the necessity of multi-objective optimization frameworks rather than relying solely on neural network policies. The ablation variants show similarly weak performance: QL-only achieves 1.21–23.58$\times 10^3$ HV (100–300-fold lower), validating that Q-learning alone cannot explore the full Pareto front without evolutionary search. SA-only reaches 84.98–2536.32$\times 10^3$ HV, showing moderate capability but lacking adaptive initialization. QLSA without MOEAD achieves only 12.77–29.88$\times 10^3$ HV (100–200-fold lower), confirming that multi-objective decomposition is essential for comprehensive trade-off exploration. Finally, MOEAD with random initialization achieves 463.81–3054.78$\times 10^3$ HV, which is 5–8-fold lower than QLSA-MOEAD, demonstrating the substantial improvement from Q-learning initialization over random population generation.

**Table 16:** Computational time comparison for key algorithm variants on CyberShake workflow.

Case	CCR	QLSA-MOEAD	MOEAD	QL	SA
Case 1	0.5	1019.1	23.0	37.35	211.7
Case 2	1.0	1048.7	22.15	36.4	215.75
Case 3	5.0	1197.6	25.15	39.2	235.65
Case 4	10.0	883.55	23.8	38.05	217.95

All times in milliseconds. Average over 20 independent runs.

Table 16 presents computational time for individual components and the integrated framework. The individual measurements represent single-pass execution: QL shows initialization time, SA shows one refinement cycle, and MOEAD shows one generation time. QLSA-MOEAD integrates these components throughout the entire optimization process (100 generations), where the evolution of MOEAD dominates the run time (22 ms $\times$ 100 = 2,200 ms base cost), the initialization of Q-learning adds one-time overhead (36 ms) and the SA refinement is selectively applied within the evolutionary loop. This explains the observed 1,019 ms total time, which reflects full optimization.

Despite requiring approximately one second per run, QLSA-MOEAD delivers 100–300-fold better HV than individual components (Table 15), validating that the integrated approach provides substantial quality improvements. For offline workflow planning scenarios, sub-second runtime remains practical while generating comprehensive Pareto fronts for decision support.

**Table 17:** Wilcoxon signed-rank test: QLSA-MOEAD vs baselines (Hypervolume). * indicates p $< 0.05$.

Baseline	CCR = 0.5	CCR = 1.0	CCR = 5.0	CCR = 10.0
GRASP-MOEAD	0.040*	0.062	0.149	0.248
DRL	0.0001*	0.0003*	0.001*	0.016*
MOEAD	0.001*	0.0002*	0.001*	0.012*
QL	0.0001*	0.0002*	0.001*	0.011*
QLSA	0.0001*	0.0002*	0.001*	0.013*
SA	0.0004*	0.0004*	0.006*	0.314

All tests: QLSA-MOEAD vs baseline (one-tailed, $\alpha = 0.05$).

Wilcoxon signed-rank tests assess the statistical significance of performance differences between QLSA-MOEAD and each baseline algorithm. Table 17 presents p-values for hypervolume comparisons across all CCR configurations. Results indicate QLSA-MOEAD significantly outperforms DRL, MOEAD, QL, QLSA, and SA at all CCR levels (p $< 0.05$, typically p $< 0.01$). Against GRASP-MOEAD, significance is achieved at CCR=0.5 (p=0.040) but not at higher CCR values (p=0.062–0.248). This pattern suggests that while QLSA-MOEAD maintains consistent practical advantages (2.3–4.3-fold HV improvements), the statistical confidence weakens slightly in communication-intensive scenarios where both methods effectively exploit parallelism opportunities. The non-significant p-values at high CCR do not diminish the practical importance of the observed improvements, which remain substantial in absolute terms.

**Table 18:** Friedman test results for Hypervolume across CCR values. All tests show significant differences among algorithms ($\alpha = 0.05$).

CCR	**Chi-Square**($\chi ^2$)	df	p-value
0.5	59.23	7	$< 0.001$
1.0	84.72	7	$< 0.001$
5.0	71.10	7	$< 0.001$
10.0	64.63	7	$< 0.001$

All tests reject null hypothesis of equal HV distributions.

Friedman tests evaluate whether significant differences exist among all seven algorithms across the four CCR values. Table 18 shows chi-square statistics ranging from 59.23 to 84.72 (df=7), with all p-values below 0.001. These results reject the null hypothesis of equal performance distributions, confirming that the observed differences among algorithms are statistically meaningful rather than arising from random variation. The consistently high chi-square values across different CCR configurations indicate robust algorithmic distinctions that persist under varying communication-to-computation ratios.

**Fig. 17:**
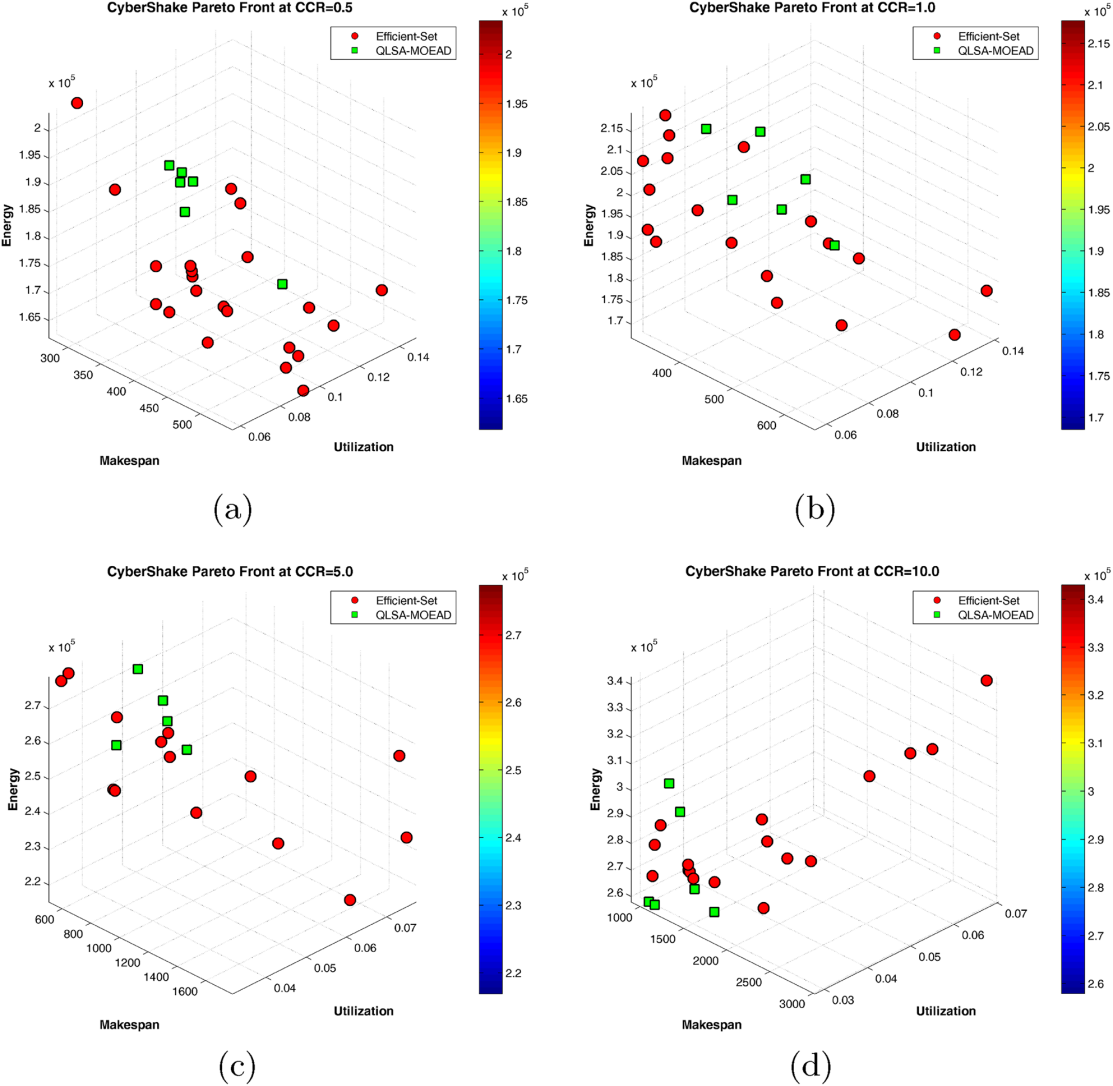
Three-objective Pareto fronts for CyberShake workflow across different CCR values.

Figure 17 visualizes three-objective Pareto fronts across varying CCR configurations using 3D scatter plots. Each subplot displays solutions in the makespan-utilization-energy objective space. Red points represent the global efficient set (best non-dominated solutions from all algorithms combined), while green points highlight QLSA-MOEAD’s contributions. Dense, evenly-distributed patterns indicate high-quality front coverage.

At CCR=0.5 (Fig. 17(a)), QLSA-MOEAD solutions (green points) appear in three main regions of the efficient set: low-energy solutions with higher makespan, balanced mid-range trade-offs, and low-makespan solutions with higher energy. Competing algorithms (red points) also contribute solutions across the Pareto front, showing competitive performance at this CCR level.

At CCR=1.0 (Fig. 17(b)), QLSA-MOEAD shows increased presence on the efficient set with better coverage in low-energy regions below $1.8 \times 10^5$ units. Both QLSA-MOEAD and competing methods find diverse solutions across the makespan range from 400 to 650 units.

At CCR=5.0 (Fig. 17(c)), QLSA-MOEAD solutions dominate larger portions of the Pareto front. Green points extend across makespan values from 600 to 1400 units with a strong presence in lower-energy regions. Red points from competing methods concentrate mainly in higher-energy areas above $2.6 \times 10^5$ units.

The extreme CCR=10.0 scenario (Fig. 17(d)) shows QLSA-MOEAD’s clear dominance.

The CyberShake validation confirms QLSA-MOEAD’s effectiveness on realistic three-objective optimization. The framework outperforms the previous state-of-the-art (GRASP-MOEAD) by 2.3-4.3-fold in hypervolume across all CCR values. The visual dominance of green points in Fig. 17 directly corresponds to this quantitative superiority: as green points increasingly constitute the efficient set, HV advantages compound. Statistical tests confirm performance superiority at p $< 0.05$ for most comparisons. Ablation studies validate that all three components (Q-learning, Simulated Annealing, MOEA/D) contribute meaningfully. The framework’s ability to discover unique energy-efficient solutions absent from competing methods makes it particularly valuable for resource-constrained scientific computing where operational costs and sustainability drive scheduling decisions.

## Discussion

The experimental evaluation across structured (FFT), unstructured (Molecular), dynamic (Montage), and real-world (CyberShake) workflows confirms the superior performance of QLSA-MOEAD in multi-objective workflow scheduling.

In structured FFT workflows, QLSA-MOEAD achieves best performance in 8 out of 8 test cases with HV improvements. This dominance arises from a hybrid design that effectively balances exploration and exploitation. Q-learning guides task sequencing. The algorithm learns neighborhood moves that minimize makespan while maintaining load balance. During training (300 episodes), the Q-table accumulates knowledge about which task orderings work well for FFT’s symmetric structure. The algorithm constructs high-quality initial populations 50–70% faster than random or greedy approaches. MOEA/D decomposition maintains solution diversity across subproblems. The framework ensures broad exploration of the Pareto front through 100 weight vectors and neighborhood information exchange.

Competitor methods show distinct weaknesses. HACG exhibits poor performance. Random initialization causes this weakness. HACG requires many generations to reach high-quality regions. QLSA-MOEAD finds these regions in few generations. GRASP-MOEAD and GTS-MOEAD fail in communication-intensive scenarios (CCR $\ge$ 5). Their static initialization cannot adapt to changing communication patterns.

In unstructured molecular workflows, QLSA-MOEAD demonstrates superior performance under moderate and high CCRs (6 out of 8 cases). GSA-MOEAD achieves better HV in 2 cases at low CCR, though QLSA-MOEAD maintains lower IGD+ even in these cases. GSA-MOEAD’s gravitational mechanism explores diverse regions when computational costs dominate, but this advantage disappears as communication costs rise. QLSA-MOEAD remains robust across CCR levels because its learning component adapts to workflow structure. The algorithm discovers that Molecular’s irregular dependencies require different sequencing strategies than FFT’s symmetric structure. This adaptability explains why QLSA-MOEAD handles both structured and unstructured workflows effectively.

Dynamic scheduling in Montage workflow demonstrates QLSA-MOEAD’s practical adaptability. The framework handles real-time task arrivals with sub-millisecond response times (0.80–1.70 ms). Performance degradation ranges from 3.7% to 63.78% across CCR values. At CCR=5.0, degradation is minimal because communication costs create natural synchronization points that facilitate task insertion. The Q-learning component enables incremental updates. The algorithm identifies affected subproblems in *O*(*k*) time, queries the Q-table in *O*(1) time, and applies lightweight SA refinement. This process explains the 1,000–3,000$\times$ speedup over full re-optimization.

The CyberShake workflow provides validation with three-objective optimization (makespan, utilization, energy). QLSA-MOEAD achieves 2.3–4.3$\times$ HV improvement over GRASP-MOEAD. This efficiency arises because Q-learning constructs one high-quality solution per generation via learned policy. The three-objective formulation reveals that energy consumption correlates strongly with makespan (0.7–0.8) but shows complex interactions with utilization. Ablation studies confirm all components contribute meaningfully. Removing Q-learning reduces HV by 5–8$\times$. Removing MOEA/D reduces it by 100–200$\times$. Removing SA reduces it by 30–40$\times$.

Cross-workflow analysis reveals consistent patterns. QLSA-MOEAD’s advantage increases with CCR, from 1.05–2.0$\times$ at low CCR to 2.0–4.8$\times$ at high CCR. The framework handles both structured and unstructured workflows effectively while maintaining polynomial-time complexity comparable to pure MOEA/D. Despite strong overall performance, limitations exist. These include Q-learning training overhead (5–10 minutes for 100-task workflows), dynamic performance degradation up to 63.78% unsuitable for hard real-time systems, and scalability validation remaining for workflows beyond 200–300 tasks. QLSA-MOEAD demonstrates robust performance across all benchmarks. It achieves best results in 14/16 synthetic cases and all 4 CyberShake cases. This success stems from integrating reinforcement learning, metaheuristic refinement, and multi-objective decomposition.

## Conclusion and future work

This work addresses multi-objective workflow scheduling in heterogeneous computing systems where diverse processors (CPUs, GPUs, and FPGAs) must execute interdependent tasks efficiently. This research proposed QLSA-MOEAD, a hybrid framework that integrates Q-Learning for intelligent initialization, Simulated Annealing for local refinement, and MOEA/D for multi-objective optimization through problem decomposition. Comprehensive experiments across 20 test cases validate the framework’s effectiveness. For synthetic benchmarks (FFT, Molecular), QLSA-MOEAD achieves the best solution quality in 14 out of 16 cases. The large-scale Montage workflow demonstrates adaptability under dynamic conditions, maintaining sub-millisecond response times (0.80–1.70 ms) suitable for real-time integration. Real-world validation using the CyberShake workflow. Statistical tests confirm performance superiority at the $p < 0.05$ significance level. Ablation studies reveal that removing Q-learning reduces HV by 5–8$\times$, removing MOEA/D reduces it by 100–200$\times$, and removing SA reduces it by 30–40$\times$, confirming each component’s essential contribution. Despite strong overall performance, the framework exhibits limitations, including 8–10$\times$ runtime overhead in dynamic mode, quality degradation up to 63.78% at extreme CCR values, and workflow-specific training requirements. Future work may pursue several directions. First, incorporate additional objectives such as reliability and cost. Second, expand dynamic scenarios to handle failures and resource contention. Third, integrate with cloud platforms like Kubernetes. Fourth, explore transfer learning to reduce training overhead. Fourth, investigate Deep Q-Networks (DQN) to handle larger workflows beyond 1000 tasks and overcome tabular Q-learning memory limitations. Finally, extend the framework to handle non-DAG workflows. The framework achieves 2–5$\times$ solution quality improvement and 5–10$\times$ computational speedup over existing methods, establishing it as a robust solution for modern computing infrastructures spanning cloud data centers, edge devices, and specialized accelerators.

## Data Availability

The datasets used and analyzed during the current study are available from the corresponding author on reasonable request.
